# Nematocyst sequestration within the family Fionidae (Gastropoda: Nudibranchia) considering ecological properties and evolution

**DOI:** 10.1186/s12983-022-00474-9

**Published:** 2022-11-16

**Authors:** Irina A. Ekimova, Olga A. Vorobyeva, Anna L. Mikhlina, Dimitry M. Schepetov, Elena V. Vortsepneva, Tatiana I. Antokhina, Vladimir V. Malakhov

**Affiliations:** 1 Invertebrate Zoology Department, Lomonosov Moscow State University, Leninskie Gori 1-12, Moscow, Russia 119234; 2 N.A. Pertsov White Sea Biological Station, Lomonosov Moscow State University, Leninskie Gori 1-12, Moscow, Russia 119234; 3 A.N. Severtsov Institute of Ecology and Evolution, Leninskiy Prosp. 33, Moscow, Russia 119071

**Keywords:** Functional morphology, Feeding modes, Adaptive radiation, Character evolution, Kleptocnidae, Chitin, Phylogeny

## Abstract

Aeolid nudibranchs are well-known for their ability to incorporate cnidarian nematocysts and use them for defense; this process is tightly linked with the feeding preferences of molluscs. As many nudibranch groups show signs of ecology-based adaptive radiation, studies of prey-based defensive mechanisms can provide valuable insight into details of nudibranch evolutionary history. The main goal of this study is to test the correlation of ecological traits, feeding mechanisms, and prey preferences with cnidosac fine morphology and to pinpoint the phylogenetic value of these traits*.* We study the cnidosac morphology in thirteen species—representatives of the main lineages within the family Fionidae *s.l.* The morphological analysis includes histological sections, transmission electron microscopy, confocal laser scanning microscopy, and scanning electron microscopy. For phylogenetic study, available molecular data from public repositories were used, and phylogenetic trees were produced based on Bayesian Inference and Maximum likelihood analysis for a concatenated dataset of three molecular markers (COI, 16S, H3). In general, fionid cnidosacs fit the common aeolid pattern, but among different species we detected a high variation in type of obtained nematocysts, their arrangement within cnidophages, and in number of cell types within cnidosacs. We report on presence of *cellules speciale* in the haemocoel of all studied species, and for the first time, we report on cells with chitinous spindles in the haemocoel of all fionids except *Eubranchus.* The function of both these cell types remains unknown. The loss of functional cnidosacs occurred at least three times within Fionidae, and in case of the genera *Phestilla, Calma,* and *Fiona,* this loss is linked to their non-cnidarian diet. The diversity of cnidosac fine structure within Fionidae *s.l.* correlates with that of the radular morphology and feeding preferences of each species. Prey shifts between cnidarian and non-cnidarian prey (both through evolutionary shifts and individual variation) rarely occur within Fionidae *s.l.;* however, microevolutionary shifts between different hydrozoan species within a single genus are more common. Cnidosac morphology demonstrates considerable resulting changes even when switching between similar hydrozoan species, or changing the feeding site on same prey species. These data indicate that cnidosac morphology likely follows microevolutionary prey shifts—in other words, it is affected by switches in prey species and changes in feeding sites with a single prey species. Thus, the cnidosac morphology may be a useful indicator when studying ecological features of particular species.

**Supplementary Information:**

The online version contains supplementary material available at 10.1186/s12983-022-00474-9.

## Introduction

Nudibranch molluscs are shell-less gastropods that have evolved a spectrum of defensive strategies. Most nudibranchs are active predators, and many groups are known for the sequestration of active biochemical compounds, organelles, and symbionts of their prey [1–6]. In most groups of nudibranch molluscs, the general feeding mode, prey preferences, and defensive mechanisms are tightly linked. The chemical defense of chromodoridid nudibranchs and some other dorid groups is supplied by secretions from dermal formations on the mantle which contain secondary metabolites of their sponge prey [7], and many chromodoridids demonstrate high specialization on particular sponge species [8]. In the Cladobranchia suborder, the genera *Phyllodesmium* and *Phestilla* exhibit close associations with their anthozoan prey and have evolved to resemble the host polyps in general appearance [4, 9–11]. Cladobranch molluscs are widely known for the ability to sequester the nematocysts of their cnidarian prey, in order to store and use them to protect themselves from predators [12–15].

The evolutionary prey shifts were believed to be the major driver of the diversification of various nudibranch groups [2]. However, further studies highlighted the discrepancies of this viewpoint using RNA-Seq-based phylogenetics and ancestral state reconstructions within major groups of Cladobranchia [16]. A strong phylogenetic correlation with prey preference is present within this group, but prey shifts at the larger scale (*a.i.* taxonomical groups at high ranks) are much more infrequent than previously thought [16]. At the same time, prey shifting at the species level likely has a primary impact on speciation within the group [16]. Accordingly, dedicated studies of the cladobranch group Dendronotidae (Dendronotoidea) showed a strong phylogenetic correlation with prey preference and feeding mode, which suggests that the evolutionary prey shift is a major speciation driver [17]. Adaptive radiation is also common within Aeolidida, and was definitively shown for the myrrhinid genus *Phyllodesmium* preying on various Octocorallia [6], and for the fionid genus *Phestilla* feeding on scleractinian corals [18]. Therefore, studies of the prey-based defensive mechanisms can provide valuable insight into the details of nudibranch evolutionary history at all taxonomic levels. Further advancement in this field would benefit greatly from dedicated studies of the ecological properties, functional morphology of the feeding apparatus, and dynamics of the prey compounds and organelles sequestration [16].

The sequestration of nematocysts (NCs) by aeolid molluscs could be a promising model [19, 20]. NCs or stinging organelles are subcellular capsules located in cnidarian cells called nematocytes, and their ability to sting is used by cnidarians for prey capture and killing, as well as for their own protection [19]. An NC contains a shaft, a tubule, and a cap at the apical end of its capsule [21]. Different types of NCs differ in shape and size, and in the morphology of their shaft and tubule [21]. In aeolids that sequester nematocysts, the obtained organelles are transported to terminal muscular sacs called cnidosacs [5, 12, 22–24]. The cnidosac is commonly subdivided into three zones of different function: the proliferation zone, the cnidophage zone, and the cnidopore zone [20, 24, 25]. Each zone is present in the cnidosacs of most aeolid nudibranchs [20] but differ in size, proportions, and cell assemblage. Several differences were found in the amount and type of obtained nematocysts and their arrangement within the cnidosac [20, 26–28], but there is no obvious correlation between sequestered nematocyst assemblage and the prey cnidom. Recent study of the fine morphological structure of cnidosacs in the species *Aeolidia papillosa* (L., 1761) (Aeolididae) indicated the cnidosac may be more complex in some species [23, 24]. In particular, these studies reported the presence of interstitial cells within the cnidophage zone. These cells were suggested to be either precursor cells (‘embryonic’ cells sensu [29]) that replace discharged cnidophages [29, 30], or supportive cells [30]. The interstitial cells in *Aeolidia papillosa* represent a unique cell type, containing a high number of vacuoles with chitinous spindles [24]. In the cnidopore zone, cnidophages are absent and the epithelial lining consists of interstitial cells only. However, it is not clear whether this feature is common for other aeolid taxa or represents an adaptation for sequestration of very long mastigophore nematocysts from anemone prey [20].

Previous studies indicated that cnidosac morphology correlates with the phylogenetic relationships within the group [20]. For instance, the monophyletic Aeolididae prey on hexacorallian groups, and most of its species sequester exclusively long and narrow mastigophores [20]. *Phestilla* nudibranchs feed on scleractinian polyps, but those with low cnidae variety, excluding nematocysts and including only spirocysts, with a few exceptions [18]. As a result, cnidosacs in these molluscs do not contain NCs [20]. Additionally, previous research has shown that the types and proportions of different sequestered NCs might vary greatly depending on the prey species chosen [22]. The radular morphology and the morphology of the buccal complex in nudibranchs is closely related to feeding mechanisms and dietary preferences [2, 17, 31–36]. As a result, molluscs that have evolved different feeding preferences, and specific adaptations in buccal armature morphology may demonstrate several differences in their sequestered NC assemblage and cnidosac morphology.

The ability of aeolid molluscs to sequester nematocysts from their prey has been discussed in a series of dedicated works (see [5] and [19] for a review). Functional cnidosacs likely represent a synapomorphic trait for Aeolidida, and the ability to obtain and store nematocysts has been lost at least three times within the group [20]. For example, the fionid genus *Phestilla* and myrrhinid *Phyllodesmium* lack kleptocnidae despite feeding on anthozoans; instead, they resemble their cnidarian prey externally, likely obtaining biochemicals from it [19, 20, 36–38]. Greenwood [39] suggested that loss of ability to sequester functional kleptocnides is likely based on the chemical and physical differences of the nematocysts themselves [2].

The family Fionidae *s.l.* (see the Material and Methods section below for a comment on taxonomic affiliation) represents a suitable model group for the study of the comparative anatomy of cnidosacs, namely to test their inter- and intraspecific or intergeneric variation and deduce their possible correlation with the feeding mechanism and diet. First of all, Fionidae *s.l.* is a large group of aeolid nudibranchs, distributed worldwide and found in all seas and oceans from the intertidal areas to deep-water environments [40–43]. The fionids are rather diverse molluscs with many species representing derived lineages, which some researchers interpret as distinct families (Eubranchidae, Tergipedidae, Cuthonidae, etc*.*) [40, 41, 44, 45]. Most of them feed on various hydrozoans [16, 31, 46], however some species have an unusual diet of fish eggs (the genus *Calma*) or stalked barnacles (the genus *Fiona*) [47–49]. Although most fionids, except *Eubranchus* and its relatives (*Leostyletus, Capellinia*), have a simple uniserial radula, its morphology varies greatly across different clades of the family, which also suggests some variation of their feeding modes [31, 50]. This implies that evolutionary prey shifts may play an important role in fionid evolution and the diversification of its main lineages—at least at the generic level [19, 40, 51]. Cnidosac morphology within the Fionidae *s.l.* also has variation [20, 26] at least in general structure, and it was suggested that the ability of nematocyst sequestration was lost at least three times (in *Phestilla*, *Calma,* and in *Fiona* and *Tergipes* clade) [20].

Our present work focuses on the comparative anatomy of cnidosacs across the main lineages of the family Fionidae, including a comprehensive study of eight genera using histological techniques, transmission electron microscopy, and confocal laser scanning microscopy. Data on cnidosac diversity were combined with studies of the feeding ecology of these molluscs. The main goal of this study is to test for a correlation of ecological traits, feeding mechanisms, and prey preferences with cnidosac fine morphology and to clarify the phylogenetic value of these traits.

## Material and methods

### Taxonomic account

The systematics of the family Fionidae *s.l.* has been recently challenged in a series of taxonomic revisions [40, 52]. Thus, we consider necessary to describe taxonomic scheme we use in the present study. In general, most researchers agreed on the close relationships of representatives of the traditional family Eubranchidae to members of families Tergipedidae, Calmidae, Fionidae, but their intergeneric affiliations remain dubious. The ‘lumping’ taxonomical scheme [40] implies there is a single family Fionidae comprising 11 genera, including *Abronica, Cuthonella, Murmania, Calma, Eubranchus, Fiona, Cuthona, Tergipes, Tergiposacca, Rubramoena* and *Tenellia*. The latter genus *Tenellia* unites most of the diversity of the traditional Tergipedidae as the most parsimonious solution, while it was highlighted that the traditional genera *Phestilla, Catriona* and *Trinchesia* required further revision [40]. Another viewpoint was suggested by Korshunova et al. [52] and updated in subsequent works [41, 44, 45 and others]. According to this approach, most tergipedid genera represent their own family, thus the Fionidae sensu Cella et al. [40] are to be split into ten families (Abronicidae, Calmidae, Cuthonellidae, Cuthonidae, Eubranchidae, Fionidae, Murmaniidae, Tergipedidae, Trinchesiidae, Xenocratenidae). Most of these families are represented by a single (sometimes monotypic) genus. Within Trinchesiidae, the authors comprised seven genera (*Catriona, Diaphoreolis, Phestilla, Rubramoena, Tenellia, Trinchesia* and *Zelentia*). However, this taxonomical scheme indicates a paraphyly of the Trinchesiidae (because of the position of the genus *Rubramoena*) and some genera like *Trinchesia, Catriona* and *Cuthona.* It also shows very poor node support and weak synapomorphies for other groups (i.e.,* Zelentia* to other Trinchesiidae, *Amphorina* to other Eubranchidae) [40, 43, 52, 53]*.* All this indicates a necessity for further revision of the group. To address these issues and provide an easy-to-follow framework in the present work, we use a curated taxonomical scheme with the monophyletic Fionidae *s.l.* (Fionidae sensu [40]) represented by 17 genera: *Abronica, Calma, Catriona, Cuthona, Cuthonella, Diaphoreolis, Eubranchus, Fiona, Murmania, Phestilla, Rubramoena, Tenellia, Tergipes, Tergiposacca, Trinchesia, Xenocratena, Zelentia.*

### Material collection

**Fig. 1:**
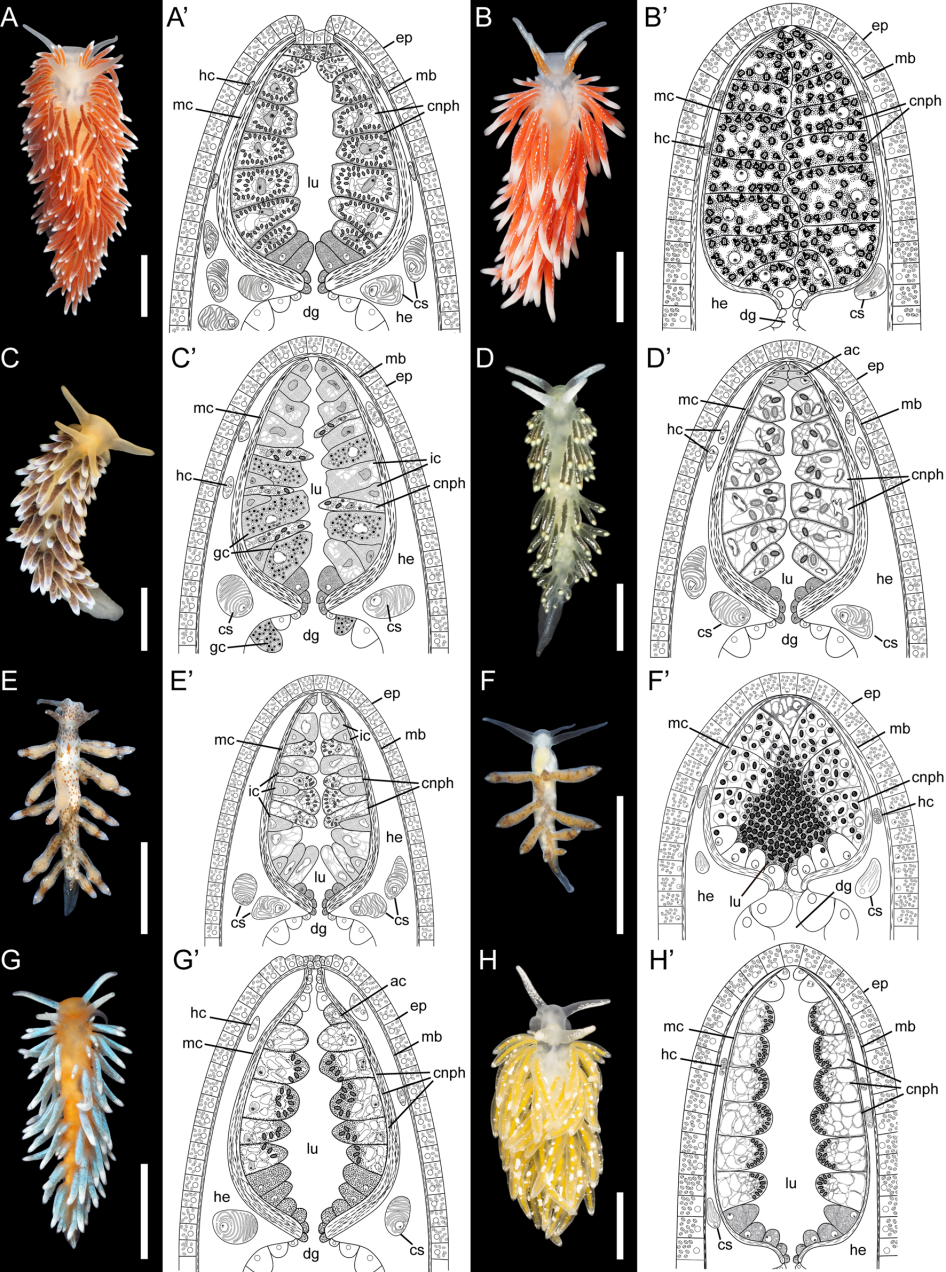
External morphology of studied nudibranch species and generalized scheme of cnidosac structure in respective species (indicated with apostrophe). **A** *Cuthona nana.* **B** *Catriona columbiana.* **C** *Cuthonella hiemalis.* **D** *Diaphoreolis viridis.* **E** *Eubranchus rupium.* **F** *Tergipes tergipes.* **G** *Trinchesia ornata.* **H** *Zelentia pustulata.* *ac* cells without NCs in cnidopore zone, *cnph* cnidophage, *cs* *cellules speciale*, *dg* digestive gland, *ep* epithelium, *gc* cells with granular compound, *hc* cells with chitinous spindles, *he* haemocoel, *ic* interstitial cells, *lu* lumen, *mb* body musculature, *mc* cnidosac musculature. Scale bars: 5 mm. Photo credits: all except C: Tatiana Antokhina, C: Alexander Semenov

The material for this study included thirteen nudibranch species of the family Fionidae *s.l.* (Fig. 1): *Catriona columbiana* (O’Donoghue, 1922), *Cuthona nana* (Alder et Hancock, 1842), *Cuthonella concinna* (Alder et Hancock, 1843), *C. hiemalis* (Roginskaya, 1987)*, C. osyoro* (Baba, 1940)*, Diaphoreolis viridis* (Forbes, 1840)*, Eubranchus malakhovi* Ekimova et al., 2021*, E. odhneri* (Derjugin et Gurjanova, 1926)*, E. pallidus* (Alder et Hancock, 1842), *E. rupium* (Møller, 1842), *Tergipes tergipes* (Forsskål, 1775), *Trinchesia ornata* (Baba, 1937), *Zelentia pustulata* (Alder et Hancock, 1854)*.* At least five specimens for each species were collected in the White Sea (*Cuthonella concinna, C. hiemalis, Diaphoreolis viridis, Eubranchus odhneri, E. rupium, Zelentia pustulata*), the Barents Sea (*Eubranchus odhneri, E. pallidus, Tergipes tergipes*) and in the Sea of Japan (*Catriona columbiana, Cuthona nana, Cuthonella osyoro, Eubranchus malakhovi, Trinchesia ornata*) during the summer seasons in 2015–2021. In most cases, the material was collected with the host hydrozoan species for the precise identification of the latter and for ecological studies (see below). In this study we used only large, fully mature specimens to avoid possible variation among different developmental stages. Before fixation, specimens were relaxed using isotonic MgCl_2_ solution (730 mOsm/kg) (1:1 with sea water) for 24 h. 10–15 cerata of each specimen were cut off and used as material for study. For the study of discharged cnidosacs, several non-relaxed specimens were disturbed with a needle, after which the cerata were cut and fixed using the above process. The number of specimens of each species studied is mentioned in Additional file 1: Table S1.

### Light microscopy

Cerata were fixed in either 2.5% glutaraldehyde in Millonig’s phosphatic buffer (pH 7.4) [54] or Bouin’s solution (2 h at 4 °C), then rinsed in Millonig’s phosphatic buffer. A postfixation was performed using 1% OsO_4_ buffered in Millonig’s phosphatic buffer for 1.5 h in the dark, after which samples were rinsed in the same buffer. Then cerata were dehydrated in a series of graded ethanol and acetone solutions, and embedded in Epon 812 resin. Series of thin sections (1 µm) were prepared with the help of LKB III and LKB V microtomes using a glass knife. Sections were stained with methylene blue (0.2%) and toluidine blue (1%) for 30–60 s and then rinsed in distilled water.

### Transmission electron microscopy (TEM)

Cerata were cut off, fixed, dehydrated, and embedded in Epon 812 resin as described in the light microscopy section. Series of ultra-thin sections (80 nm) were prepared with a Leica EM UC6 ultramicrotome using a Ultra 45 diamond knife (Diatome, Switzerland). The sections were stained with uranyl acetate (1%, 40 min, 37 °C) and lead citrate (10 min). The sections were analyzed using JEM-1011 (JEOL, Japan) and JEM-1400 (JEOL, Japan) transmission electron microscopes.

### Confocal laser scanning microscopy (CLSM)

For CLSM, cerata were fixed in 4% paraformaldehyde (PFA; Fluka, Germany) in phosphate-buffered saline (PBS; Fluka, Germany) at 4 °C for 24 h, rinsed thrice for 30 min in 0.1 M PBS containing 0.1% Triton X-100 (Ferak, Berlin, Germany; PBS-TX), then incubated in blocking solution (1% BSA, 0.1% cold fish skin gelatin (Sigma), 2.5% Triton X-100, 0.05% Tween 20, 0.05% sodium azide in PBS) thrice for 8 h. Samples were then stained for 48 h (4 °C) with anti-acetylated α-tubulin mouse-raised primary antibodies (cat. no. T6793, Sigma-Aldrich) for tubulin visualization in cilia and neural elements. Antibodies were diluted in blocking solution according to manufacturer protocols. After incubation, samples were washed thrice in blocking solution for 1 h and incubated for 48 h at 4 °C with Donkey Anti-Mouse IgG secondary antibodies labeled with Alexa Fluor 488 (Molecular Probes, Cat #A21202). The dilution was 1:500–1:1000 according to manufacturer protocol. The samples were then stained for 4–8 h with Alexa Fluor 647 phalloidin (1:100; Molecular Probes, Cat #A22287) for actin labeling, with Propidium iodide nuclear stain for 1 h at 4 °C, and with Calcofluor White Dye (1 h, 4 °C) for specific labelling of the amorphic chitin. All stains were diluted in PBS. After staining, the samples were rinsed in PBS for 30 min, cleared in graded isopropyl alcohol series (30 s for each stage) and Murray’s clear (one-part benzyl alcohol with two parts benzyl benzoate, stained for 1 m, three times), then mounted using Murray’s clear. The samples were analyzed using a Nikon A1R-A1 confocal microscope (Nikon Corporation, Tokyo, Japan). Z-projections and optical Z-sections were generated using the programs NIS-Elements D4.50.00 (Nikon) and Image J V.1.43 (https://imagej.nih.gov/ij/) and processed in Adobe Photoshop CS5 Extended v. 12.0.3 × 32 (Adobe Systems, USA). Some samples (several specimens of the species *Cuthonella concinna, Eubranchus odhneri, Cuthona nana, Catriona columbiana*) showed positive NC staining with the Calcofluor White Dye, where staining was negative in other samples. This is likely because of the presence of chitin in the walls and tubules of NCs, as was previously mentioned for *Aurelia* and *Hydra* nematocysts [55]. We are not sure whether the absence of Calcofluor White Dye signal is a result of low fluorescence emission due to the thickness of sample, or of chitin absence in NCs. However, this is not important to the conclusions garnered in this study.

### Scanning electron microscopy (SEM)

For the SEM study of buccal armature general morphology, we extracted the radular apparatus and jaw plates from all studied species, incubated them in proteinase K solution (diluted in buffer 1:10) for 10 h at 60 °C. They were then rinsed in distilled water, air-dried, mounted on an aluminum stub, and sputter-coated with platinum-palladium. The samples were observed using a Camscan S2 scanning electron microscope (Camscan Electron Optics Ltd., England), or EVO-40 (Zeiss, Germany), or JSM7000 (JEOL, Japan).

### Nematocyst identification

The type of sequestered NCs was identified using two methods: (1) analysis of the thin and ultrathin sections of the cnidosac, and (2) analysis of NCs in discharged cnidosacs using the Transmission Detector Analyzer option in the confocal laser scanning microscope Nikon A1R-A1 (Nikon Corporation, Tokyo, Japan). For this purpose, we followed the classification and NC descriptions provided by Östman [21, 56]. In both identification methods, the three characters were used: (1) the shape of the NC; (2) the presence, size, and shape of the shaft, and (3) the spine pattern of the shaft and the tubule (which is clearly visible in both TEM and CLSM). Due to the restrictions of this methodology, we did not identify types of NCs (e.g., p-/b-mastigophores, homotrichous/heterotrichous euryteles, etc.) as that would require SEM studies of discharged NCs. The precise number of specimens studied is shown in Table S1 (columns TEM + CLSM).

### In vivo observations

Adult specimens of *Cuthona nana* (two specimens), *Cuthonella concinna* (five specimens), *Cuthonella hiemalis* (two specimens)*, Diaphoreolis viridis* (three specimens), *Tergipes tergipes* (three specimens) and *Zelentia pustulata* (two specimens) were used in laboratory in vivo observations. These were preceded by extensive studies of the feeding behavior of these species underwater, including identification of their host hydrozoan and detection of the nudibranch’s position on it. Additionally, we observed the feeding behavior of *Catriona columbiana* and *Trinchesia ornata* during sampling.

The specimens were kept starved in the tank with filtered sea water for 48 h at 4 °C in the cases of *Cuthonella concinna*, *Cuthonella hiemalis, Diaphoreolis viridis*, *Tergipes tergipes* and *Zelentia pustulata,* and for 24 h at 8 °C in the case of *Cuthona nana.* They were then placed into an aquarium with the prey species. The feeding process of *C. hiemalis* and *D. viridis* was photographed at a speed of 1 frame per second, using a Nikon D-3400 camera with a Nikon AF-S VR Micro-Nikkor 105 mm f/2.8G IF-ED. The time-lapse video was created at a 24 fps frame rate using Sony Vegas Pro 12.0 software (Sony Creative Software, Middleton, US). The feeding process of *C. nana*, *C. concinna, T. tergipes* and *Z. pustulata* was filmed using a LabCam Pro Microscope Adapter for iPhone (LabCam™, iDu Optics, Detroit, US) mounted on a Olympus SZ51 stereomicroscope (Olympus Corporation, Tokio, Japan). Separate frames from the video showing different stages of the feeding process were selected using Sony Vegas Pro 12.0 software (Sony Creative Software, Middleton, US).

### Phylogenetic methods

For mapping the cnidosac characters, features of buccal armature, and diet preferences on the current phylogenetic reconstruction of the family Fionidae *s.l.,* we used molecular data of three markers (COI, 16S and H3) that are publicly available in the NCBI database (see Table S2 for GenBank accession numbers). Sequences were aligned with the MUSCLE [57] algorithm in MEGA 7 [58]. Additionally, all protein-coding sequences were translated into amino acids to verify reading frames and check for stop-codons. To check saturation, the total number of pairwise differences (transitions and transversions) for all specimens (including those in the outgroup), were plotted against uncorrected p-distances. For the COI and H3 fragments, saturation was further examined separately for the first, second and third codon positions. Indel-rich regions of the 16S alignment were identified and removed in Gblocks 0.91b [59] with the least stringent settings. Sequences were concatenated by a simple biopython script following Chaban et al. [60]. Phylogenetic reconstructions were conducted for the concatenated multi-gene partitioned datasets. The best-fit nucleotide evolution model for the MrBayes phylogeny reconstruction method were selected in ModelTest-NG v0.1.7 [61, 62]: GTR + G + I for the COI alignment, HKY + G + I for the 16S alignment, and GTR + G for the H3 alignment. Multi-gene analyses were done by applying evolutionary models separately to partitions representing single markers. The Bayesian phylogenetic analyses and estimation of posterior probabilities were performed in MrBayes 3.2 [63]. Markov chains were sampled at intervals of 500 generations. The analysis was initiated with a random starting tree and ran for 10^7^ generations. Maximum likelihood phylogeny inference was performed in the HPC-PTHREADS-AVX option of RaxML HPC-PTHREADS 8.2.12 [64] with 1000 pseudoreplicates. The same models as in the Bayesian analysis were used for each partition. Bootstrap values were placed on the best tree found with SumTrees 3.3.1 from the DendroPy Phylogenetic Computing Library 3.12.0 [65]. Final phylogenetic tree images were rendered in FigTree 1.4.0 and further visually modified in Adobe Illustrator CS 2015.

## Results

### General cnidosac morphology in Fionidae *s.l.*

**Fig. 2:**
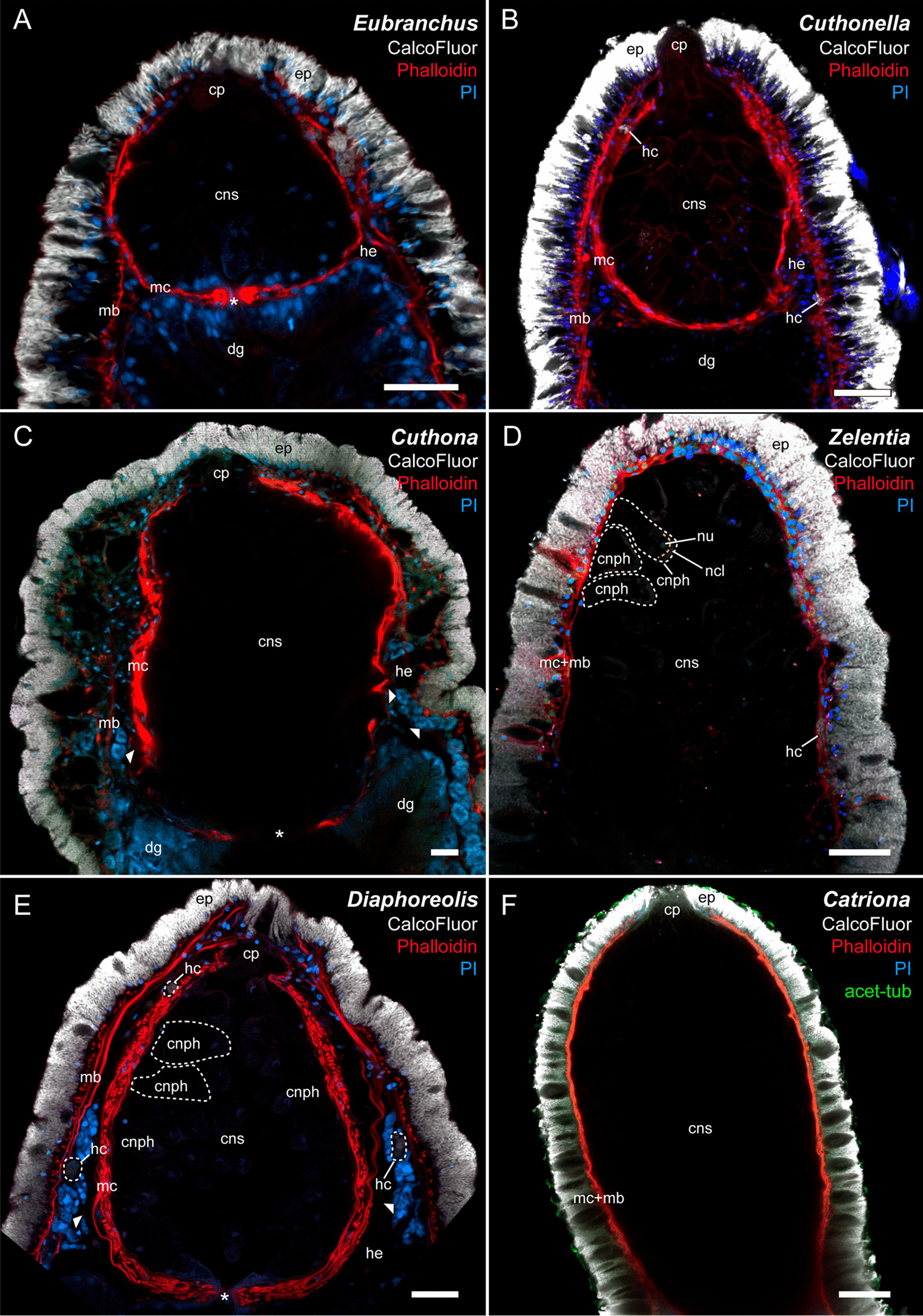
Longitudinal optical section of cnidosac in different Fionidae species (CLSM). **A** *Eubranchus rupium*; **B** *Cuthonella hiemalis*; **C** *Cuthona nana*; **D** *Zelentia pustulata,* proximal end of cnidosac is not seen due to its large size, white dotted lines indicate cnidophages, yellow dotted line indicates NCs layer; **E** *Diaphoreolis viridis,* white dotted lines in cnidosac indicate cnidophages, white dotted lines in haemocoel indicates cells with chitinous elements*;* **F** *Catriona columbiana,* proximal end of cnidosac is not seen due to its large size. *cnph* cnidophage, *cns* cnidosac, *cp* cnidopore, *dg* digestive gland, *ep* epithelium, *hc* haemocoel cells with chitinous spindles, *he* haemocoel, *mb* body musculature, *mc* cnidosac musculature. White arrowheads indicate *cellules speciale*, star—cnidosac entrance (where applicable). Scale bars: 20 µm

**Fig. 3:**
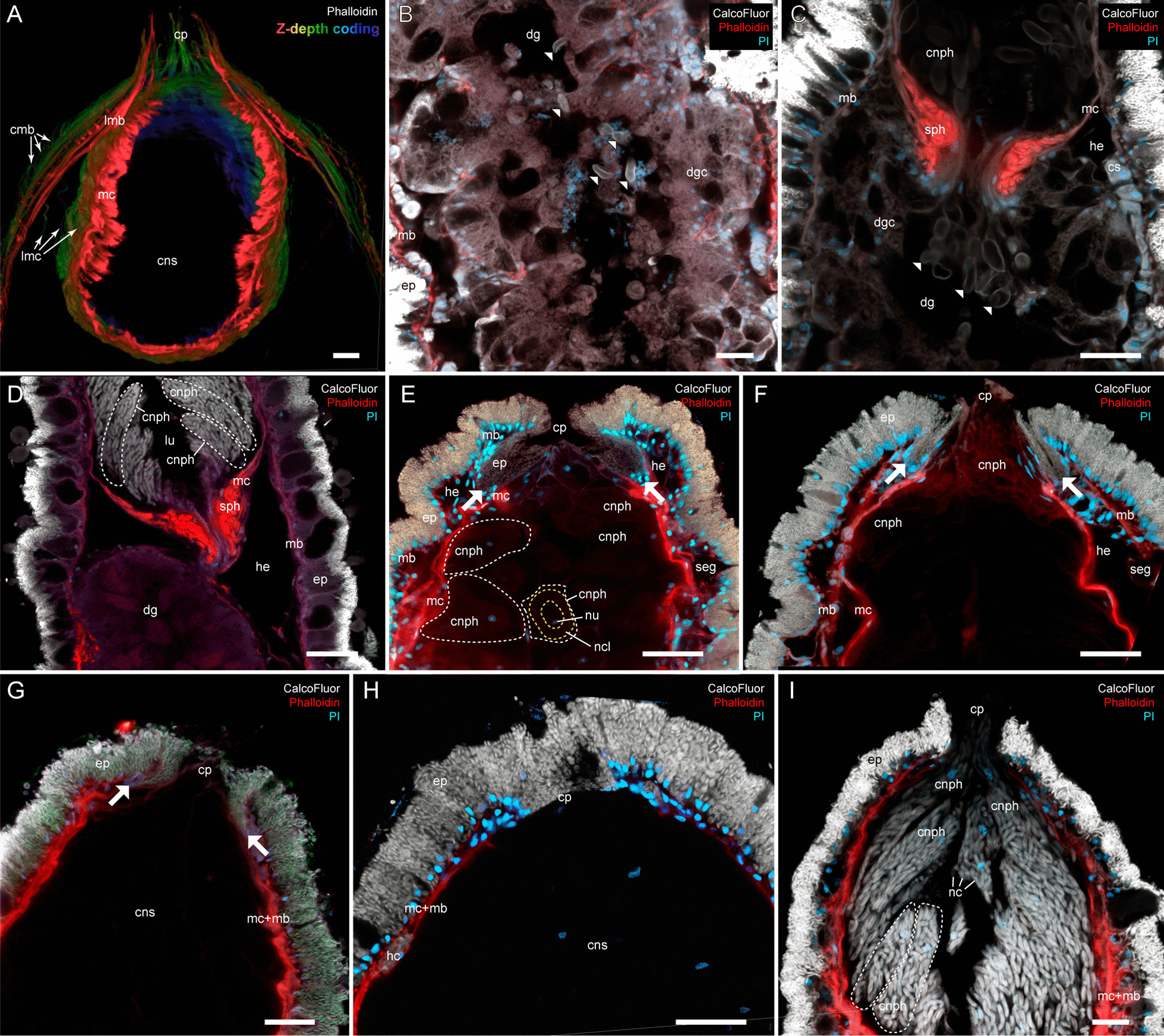
Musculature, digestive gland diverticula, cnidosac entrance and cnidopore in different Fionidae species (CLSM). **A** *Diaphoreolis viridis,* 3D-reconstruction of musculature of ceratal distal part. **B** *Cuthonella concinna,* optical longitudinal section, digestive gland diverticula showing intact nematocysts in digestive gland lumen (white arrowheads), the brightness/contrast is enhanced to make nematocysts visible among mollusc tissues. **C** *Cuthonella concinna,* optical longitudinal section, cnidosac entrance showing intact nematocysts in digestive gland lumen (white arrowheads), the brightness/contrast is excessive to make nematocysts visible among mollusc tissues. **D** *Eubranchus odhneri,* optical longitudinal section, cnidosac entrance, white dotted lines indicate cnidophages with NCs. **E** *Cuthona nana,* optical longitudinal section, cnidopore with invagination of epidermal layer closely adjacent to cnidophages (borders are indicated with white arrows), white dotted lines indicate cnidophages, yellow dotted lines indicate NCs layer. **F** *Cuthona nana,* optical longitudinal section, discharged cnidosac, cnidopore with ejected cnidophages containing nematocysts (borders are indicated with white arrows). **G** *Catriona columbiana,* optical longitudinal section, cnidopore with invagination of epidermal layer closely adjacent to cnidophages (borders are indicated with white arrows). **H** *Zelentia pustulata,* optical longitudinal section, cnidopore. **I** *Eubranchus odhneri,* optical longitudinal section, cnidopore with ejected cnidophages containing nematocysts, white dotted lines indicate cnidophages with NCs. *cmb *circular musculature of body, *cnph* cnidophage, *cns* cnidosac, *cp* cnidopore, *cs* *cellules speciale,* *dg* digestive gland, *dgc* digestive gland cells, *ep* epithelium, *hc* haemocoel cell with chitinous spindles, *he *haemocoel, *lmb* longitudinal musculature of body, *lmc* longitudinal musculature of cnidosac, *lu* lumen, *mb* body musculature, *mc* cnidosac musculature, *nc* NCs, *ncl* NCs layer within cnidophage, *nu* nucleus, *seg* subepidermal mucus gland, *sph* muscular sphincter of cnidosac. Scale bars: 20 µm

The cnidosac is a continuation of the digestive diverticulum, and in all cases there is only one cnidosac per ceras (Figs. 1, 2, 3). It is formed by the longitudinal (outer) and the circular (inner) musculature layers, which connect to the corresponding layers of the ceratal musculature in the cnidopore area (the circular layer is adjacent to the epidermis, and the longitudinal layer is to the haemocoel) (Figs. 1, 2A). The longitudinal musculature of the cnidosac also shows several connections to ceratal musculature crossing the haemocoel in the middle parts of the cnidosac (Fig. 2A). In several species, the cnidosac musculature is thin and the layers are hardly distinguishable (Fig. 2D, F).

**Fig. 4:**
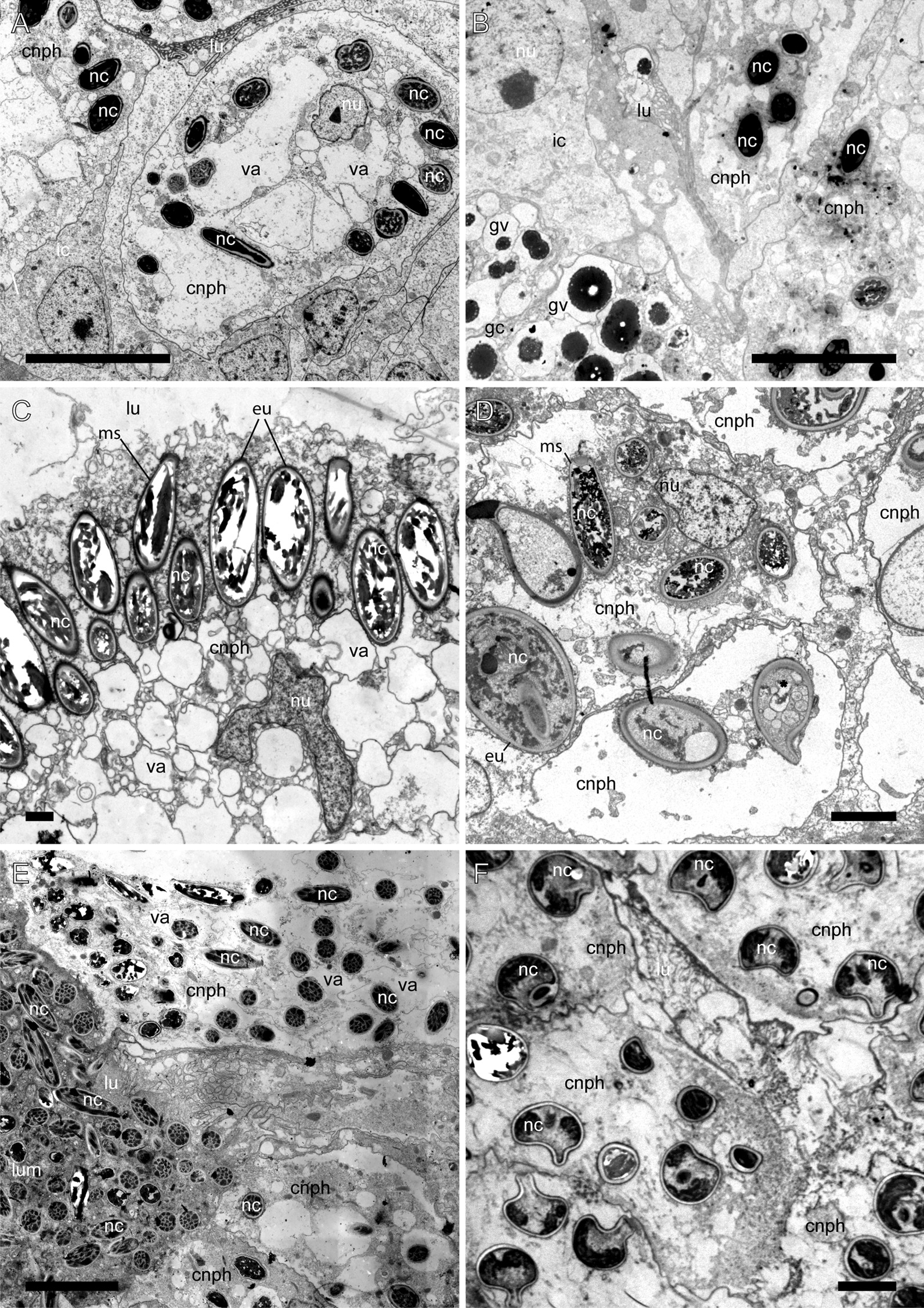
Different arrangement of nematocysts within cnidophages of different Fionidae species (TEM). **A** Mastigophores in *Eubranchus rupium.* **B** Mastigophores in *Cuthonella hiemalis.* **C** Euryteles and mastigophores in *Zelentia pustulata.* **D** Euryteles and mastigophores in *Diaphoreolis viridis.* **E** Mastigophores *Tergipes tergipes.* **F** Stenoteles in *Catriona columbiana.* *cnph* cnidophage, *eu* euryteles, *gv* vacuoles with unidentified granular content, *ic* interstitial cell, *lu* lumen, *ms* mastigophores, *nc* nematocyst, *nu* nucleus, *va* vacuole. Scale bars: **A**, **B**, **E**, **F**—10 µm, **C**—2 µm, **D**—5 µm

**Fig. 5:**
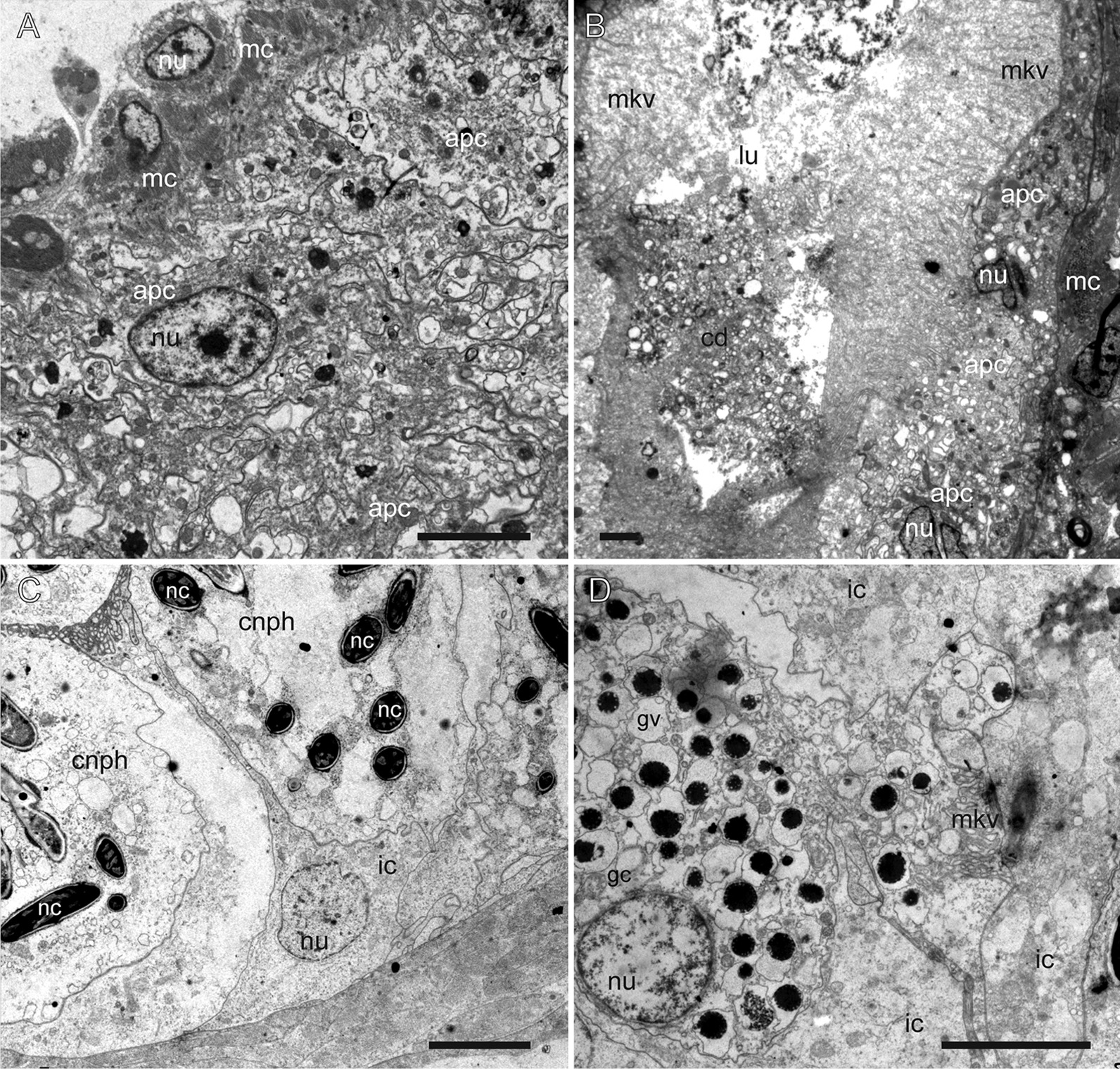
Additional cell types in cnidosacs of different Fionidae species (TEM). **A** *Diaphoreolis viridis.* **B** *Trinchesia ornata.* **C** *Eubranchus rupium.* **D** *Cuthonella hiemalis.* *apc* degraded cells, *cd* cell debris, *cnph* cnidophage, *gc* cell with granular content, *gv* vacuoles with unidentified granular content, *ic* interstitial cell, *lu* lumen, *mc* cnidosac musculature, *mkv* microvilli, *nc* nematocyst, *nu* nucleus. Scale bars: **A**, **C**, **D**—5 µm, **B**—2 µm

The cnidosac is subdivided into three functional areas: the proliferation zone, the cnidophage zone, and the cnidopore zone (Figs. 1, 2, 3). The cnidosac entrance connects the digestive gland to the cnidosac lumen (Fig. 3D). In several species, we detected a large number of intact nematocysts (NCs) in the digestive gland diverticulum in the basal (Fig. 3B) and apical parts of the ceras (Fig. 3C). The cnidosac entrance is surrounded by a strong, muscular sphincter (Fig. 3C, D). The proliferation zone lies next to the cnidosac entrance (Fig. 2). The cnidophage zone occupies the main cnidosac volume and contains cnidophages with NCs of different types (Figs. 1, 4) that depend on the mollusc’s diet (Table S3). Overall, four NC types were found: euryteles, stenoteles, mastigophores, and isorhizas (Fig. 4, Additional files 3, 4: Tables S3, S4). NC arrangement within cnidophages varies greatly among the representatives of different genera (Fig. 4, Additional file 7–15: Figs. S1–9, Additional file 4: Table S4). In several species (*Cuthona nana*, *Catriona columbiana*, *Tergipes tergipes, Zelentia pustulata*), the cnidosac lining consists of one cell type (cnidophages) (Fig. 4E, Additional file 7: Fig. S1B, Additional file 8: Fig. S2D, Additional file 15: Fig. S9E); in other species (i.e., representatives of the genus *Cuthonella, Eubranchus rupium*), additional cell types like interstitial cells and cells with various inclusions may be found (Fig. 5C, D). The cnidosac lumen is well-developed in several species (*Cuthona nana*, *T. tergipes*, *Z. pustulata*) and almost absent in others (*Catriona columbiana*, *E. rupium*) (Fig. 1). In some species (*Z. pustulata*, *E. odhneri*), the cnidophage zone continues to the cnidopore, which is referred as the “simple cnidopore” according to the terminology suggested in [20] (Fig. 3H, I). In other species, several modifications are found: the cnidopore is lined by cells without nematocysts (*D. viridis*, *T. ornata*, *E. rupium*, *Cuthonella hiemalis*) (Fig. 5), or is demarcated by an invagination of the epidermis (*Cuthona nana*, *Catriona columbiana*) (Fig. 3E–G).

**Fig. 6:**
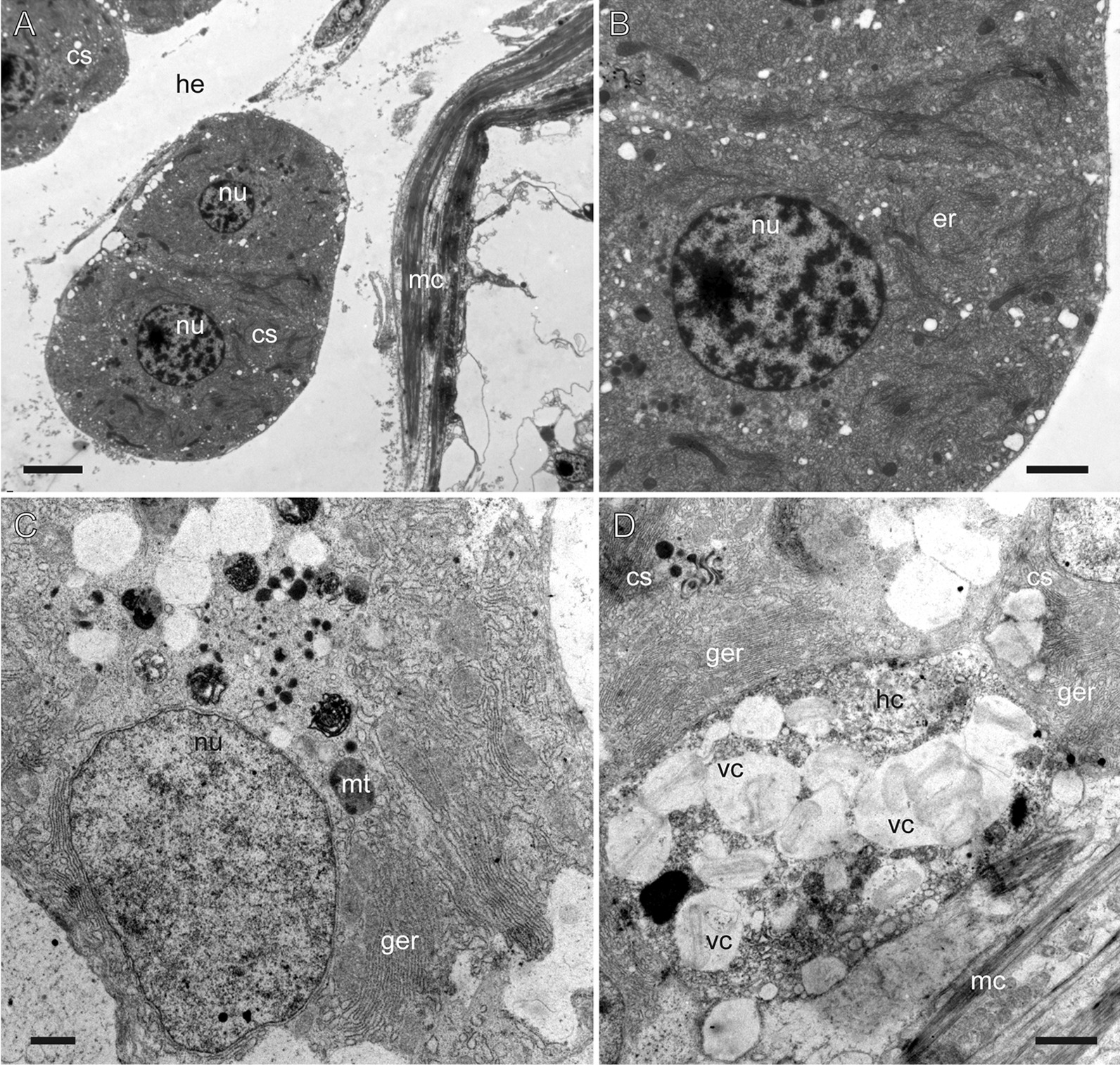
Haemocoel cells (TEM). **A**, **B** *Zelentia pustulata, cellules speciale.* **C** *Cuthonella hiemalis, cellule speciale.* **D** *Cuthonella hiemalis, cellules speciale* and cell with chitinous spindles*.* *cs* cellule speciale, *ger* granular reticulum, *er* reticulum (unidentified), *he* haemocoel, *hc* haemocoel cell with granular chitin, *mc* cnidosac musculature, *mt* mitochondria, *nu* nucleus, *vc* vacuoles with chitinous spindles. Scale bars: **A** 3 µm, **B**, **C** 1 µm, **D** 2 µm

The haemocoel near the cnidosac area of Fionidae *s.l.* species shows several specific features. In all cases it contains a unique cell type–the so-called *cellules speciale *sensu Edmunds [26], which have a cytoplasm that shows positive staining with nucleic dye Propidium iodide (Fig. 2C, E). At the ultrastructural level they have a large nucleus, and the cytoplasm is filled with a granular endoplasmic reticulum (Fig. 6A–C). In all studied species, except representatives of the genus *Eubranchus,* we also detected cells containing vacuoles with chitinous spindles in the haemocoel (Figs. 2, 6D).

**Fig. 7:**
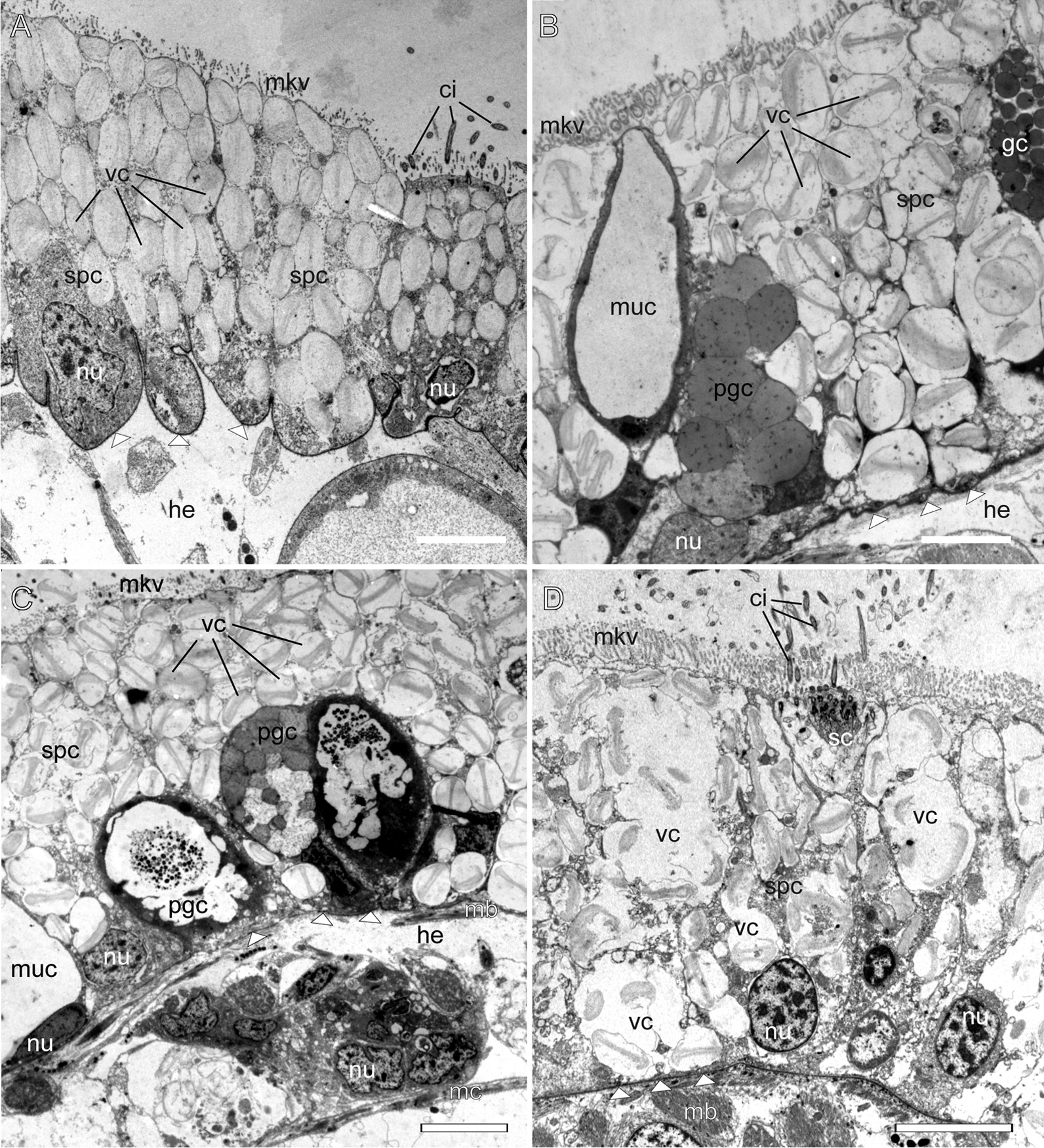
Epidermis in different Fionidae species (TEM). **A** *Eubranchus rupium.* **B** *Catriona columbiana.* **C** *Zelentia pustulata.* **D** *Diaphoreolis viridis.* *ci* cilia, *gc* cell with granular compound, *he* haemocoel, *mb* body musculature, *mc* cnidosac musculature, *muc* mucous cell, *mkv* microvilli, *nu* nucleus, *pgc* pigment cell, *sc* sensory cell, *spc* supportive cells, *vc* vacuoles with chitinous spindles. White triangles indicate epidermal basal lamina. Scale bars: 5 µm

The epidermis shows a typical structure for cladobranch molluscs. It is underlined by a wrinkled, thick basal lamina (Fig. 7). The epidermis comprises supportive cells, mucous cells, cells with different types of granular electron-dense compounds, and sensory cells. Supportive cells form a dense layer, their cytoplasm has numerous vacuoles carrying chitinous spindles (Fig. 7). Mucous cells contain large vacuoles with loose electron-transparent compounds, and occupy a subepidermal position in several species (Figs. 2C, 7A). Cells with electron-dense granules are common in the epidermis (Fig. 7B, C). Sensory cells are rare and possess a bunch of cilia (Fig. 7D).

### Cnidosac ultrastructure in studied species

All studied specimens of each species show no valuable intraspecific variation in the cnidosac morphology.

#### *Catriona columbiana* (Additional file 7: Fig. S1)

Thin-walled cnidosac (Fig. 2F, Additional file 7: Fig. S1B). Muscle layers poorly developed (up to 2 µm in thickness). Cnidosac lining consists of cnidophages only (Additional file 7: Fig. S1B, C). Cnidophages large, voluminous, containing numerous NCs (Additional file 7: Fig. S1B, C). NCs of single type, stenoteles, arranged at periphery of cells, not enclosed into vacuole, oriented by cap to cell membrane (Fig. 4F, Additional file 7: Fig. S1D, E, G, H). Nucleus with single conspicuous dense nucleolus. Cytoplasm electron-transparent, with few vesicles and electron-dense granules (Additional file 7: Fig. S1C, D). Lumen small with few microvilli and cell processes. *Cellules speciale* present in haemocoel. Chitinous spindles present in haemocoel. Cnidopore simple (Fig. 2F).

#### *Cuthona nana* (Additional file 8: Fig. S2)

Muscle layers well-developed (up to 9 µm in thickness) (Fig. 2C, Additional file 8: Fig. S2B). Cnidosac lining consists of cnidophages only (Additional file 8: Fig. S2B, D). Cnidophages large, voluminous, containing numerous NCs (Additional file 8: Fig. S2B, D). NCs of single type, microbasic euryteles, not enclosed into vacuole, arranged in circle around nucleus and most cell organelles (Fig. 3E, Additional file 8: Fig. S2B, D, E). Peripheric cytoplasm electron-transparent, with few vesicles and electron-dense granules (Additional file 8: Fig. S2D). Nucleus with single conspicuous dense nucleolus (Fig. 3E). Lumen small with few microvilli and cell processes. *Cellules speciale* present in haemocoel (Additional file 8: Fig. S2H). Chitinous spindles present in haemocoel. Cnidopore complex with invagination of epidermal layer connected with cnidosac epithelium by basal lamina (Fig. 3E, F).

#### *Cuthonella hiemalis* (Additional file 9: Fig. S3)

Muscle layers well-developed (up to 6 µm in thickness) (Fig. 2B, Additional file 9: Fig. S3I). Three cell types in cnidophage zone lining (cnidophages, interstitial cells, and cells with inclusions) (Fig. 5D, Additional file 9: Fig. S3F, G, H). Cnidophages elongated, containing few NCs (Additional file 9: Fig. S3G, H). NCs of two types—mastigophores and isorhizas—arranged irregularly, enclosed in vacuoles (Additional file 9: Fig. S3G, H). Nucleus with single conspicuous dense nucleolus (Additional file 9: Fig. S3). Cytoplasm electron-dense, containing numerous granules, vesicles, and large vacuoles with electron-transparent content (Additional file 9: Fig. S3H). Cells with inclusions containing numerous vacuoles with electron-dense contents (Additional file 9: Fig. S3F, G, I). Vacuolar content with solid center and porous periphery (Additional file 9: Fig. S3I). These cells bear numerous microvilli (Fig. 5D). Interstitial cells contain few vesicles and electron-dense granules, nucleus without obvious nucleolus (Fig. 5D, Additional file 9: Fig. S3F). Lumen small with microvilli and cilia (Figs. 4B, 5D). *Cellules speciale* present in haemocoel (Additional file 9: Fig. S3J, K). Chitinous spindles present in haemocoel (Fig. 2B, Additional file 9: Fig. S3E). Cnidopore simple (Fig. 2B).

#### *Cuthonella concinna* and *Cuthonella osyoro*

Both species show similar cnidosac morphology to *C. hiemalis,* but in *C. concinna* NCs type differs, containing euryteles and mastigophores with different capsule proportions (Fig. 3C, white arrowheads).

#### *Diaphoreolis viridis* (Additional file 10: Fig. S4)

Muscle layers well-developed (up to 8 µm in thickness) (Fig. 2E, Additional file 10: Fig. S4D). Single cell type (cnidophages) (Additional file 10: Fig. S4D, E). Cnidophages elongated, voluminous, containing few NCs per cell (Fig. 2E, Additional file 10: Fig. S4D, E). NCs of different types, euryteles, mastigophores, and isorhizas, most concentrated in apical part of cell, some in other cytoplasm parts (Figs. 4D, Additional file 10: Fig. S4D-G). NCs enclosed in vacuoles (Fig. 4D, Additional file 10: Fig. S4D, E). Nucleus with single conspicuous dense nucleolus (Additional file 10: Fig. S4D). Cytoplasm electron-transparent, containing numerous large, voluminous vacuoles with electron-transparent content, and many small electron-dense vesicles and small vacuoles surrounding nematocysts (Additional file 10: Fig. S4F). Lumen small with microvilli and cilia. *Cellules speciale* present in haemocoel (Fig. 2E). Chitinous spindles present in haemocoel (Fig. 2E). Cnidopore area forms narrow channel, lined by NC-free cells with short microvilli (Fig. 5A, Additional file 10: Fig. S4C).

#### *Eubranchus pallidus* (Additional file 11: Fig. S5)

Muscle layers well-developed (up to 6 µm in thickness) (Additional file 11: Fig. S5B, D). Two cell types (cnidophages and interstitial cells) (Additional file 11: Fig. S5C). Cnidophages voluminous, containing many NCs per cell (Additional file 11: Fig. S5B, C). NCs of two types, mastigophores and isorhizas, most arranged irregularly in apical cell part adjacent to lumen, some oriented by cap to membrane (Additional file 11: Fig. S5B, C, G). NCs enclosed in vacuoles (Additional file 11: Fig. S5E). Nucleus under NC layer (Additional file 11: Fig. S5C). Nucleus with single conspicuous dense nucleolus (Additional file 11: Fig. S5C). Cytoplasm electron-dense, containing numerous vacuoles with electron-transparent compound and many small electron-dense vesicles and small vacuoles surrounding NCs (Additional file 11: Fig. S5C). Interstitial cells with inclusions, containing numerous vacuoles with electron-dense contents (Additional file 11: Fig. S5C). Lumen large with microvilli and cilia (Additional file 11: Fig. S5C, G). *Cellules speciale* present in haemocoel. Chitinous spindles not found in haemocoel. Cnidopore area formed by interstitial cells (Additional file 11: Fig. S5G).

#### *Eubranchus rupium* (Additional file 12: Fig. S6)

Muscle layers not well-developed (up to 2 µm in thickness) (Fig. 2A, Additional file 12: Fig. S6D). Two cell types (cnidophages and interstitial cells). Cnidophages elongated, voluminous, containing few NCs per cell (Fig. 4A). NCs of two types, mastigophores and isorhizas, arranged irregularly in apical cell part adjacent to lumen, enclosed in vacuoles (Additional file 12: Fig. S6H). Nucleus under NC layer (Additional file 12: Fig. S6G). Nucleus with single conspicuous dense nucleolus (Additional file 12: Fig. S6G). Cytoplasm electron-transparent, containing numerous large, voluminous vacuoles with electron-transparent compounds and many small electron-dense vesicles and small vacuoles surrounding NCs (Additional file 12: Fig. S6G). Interstitial cells with inclusions, containing numerous vacuoles with electron-dense contents (Additional file 12: Fig. S6F, G). Lumen small with microvilli and cilia (Additional file 12: Fig. S6E). *Cellules speciale* present in haemocoel (Additional file 12: Fig. S6B). Chitinous spindles not found in haemocoel. Cnidopore area formed by interstitial cells (Additional file 12: Fig. S6B).

#### *Eubranchus odhneri* and *E. malakhovi*

Full description of cnidosac morphology in these two species given in Ekimova et al. [53] (present study using CLSM and TEM confirms these data). Cnidophages large, voluminous cells containing numerous NCs (mastigophores), enclosed into vacuoles. Cnidophage cytoplasm highly vacuolated in *E. malakhovi* or electron-transparent without vacuoles in *E. odhneri*. *Cellules speciale* present in haemocoel. Chitinous spindles not found in haemocoel. Cnidopore simple.

#### *Tergipes tergipes* (Additional file 13: Fig. S7)

Muscle layers well-developed (up to 5 µm in thickness) (Additional file 13: Fig. S7B, C). Cnidosac lining consists of cnidophages only (Additional file 13: Fig. S7C). Cnidophages large, voluminous, containing numerous NCs (Fig. 4C). NCs of two types, mastigophores and isorhizas, arranged irregularly, enclosed in large vacuoles (Additional file 13: Fig. S7B–E). Nucleus in basal cell part located close to musculature layers. Nucleus with single conspicuous dense nucleolus (Additional file 13: Fig. S7D). Lumen very large, filled with numerous NCs and intercellular matrix (Additional file 13: Fig. S7C, D). *Cellules speciale* present in haemocoel (Additional file 13: Fig. S7F). Chitinous spindles present in haemocoel (Additional file 13: Fig. S7F). Cnidopore simple.

#### *Trinchesia ornata* (Additional file 14: Fig. S8)

Muscle layers well-developed (up to 5 µm in thickness) (Additional file 14: Fig. S8B). Cnidosac lining with single cell type (cnidophages) (Additional file 14: Fig. S8B). Cnidophages elongated, voluminous, containing few NCs per cell (Additional file 14: Fig. S8B, C). NCs of single type, mastigophores, most concentrated in apical part, some in other cytoplasm parts (Additional file 14: Fig. S8B, C). NCs not enclosed in vacuoles (Additional file 14: Fig. S8E, F). Nucleus with single conspicuous dense nucleolus. Cytoplasm electron-dense, containing numerous vacuoles with electron-transparent content and many small electron-dense vesicles and small vacuoles surrounding nematocysts (Additional file 14: Fig. S8B, C). Lumen small with microvilli and cilia. *Cellules speciale* present in haemocoel. Chitinous spindles present in haemocoel. Cnidopore area forms narrow channel lined by degraded NC-free cells with long microvilli (Fig. 5B, Additional file 14: Fig. S8D, G).

#### *Zelentia pustulata* (Additional file 15: Fig. S9)

Thin-walled cnidosac (Fig. 2D). Muscle layers poorly developed (up to 1.5 µm in thickness) (Additional file 15: Fig. S9E). Cnidosac lining consists of cnidophages only (Additional file 15: Fig. S9E). Large cnidophages containing numerous NCs (Additional file 15: Fig. S9E, F). Adjacent to muscular layer of cnidosac cnidophages contain large electron-transparent vacuoles, their size decreases toward the lumen (Additional file 15: Fig. S9E, F). NCs form compact layer near cell membrane adjacent to lumen (Fig. 4C, Additional file 15: Fig. S9E). NCs of two types, mastigophores and euryteles. NCs not enclosed into vacuole, oriented by cap to membrane (Additional file 15: Fig. S9F, G). Nucleus under NC layer (Additional file 15: Fig. S9F), with single conspicuous dense nucleolus. Lumen large, electron-transparent, with few microvilli and cilia (Additional file 15: Fig. S9E). *Cellules speciale* present in haemocoel (Fig. 6A, B, Additional file 15: Fig. S9D). Chitinous spindles present in haemocoel (Figs. 2D, 3H). Cnidopore simple.

## *Feeding mechanisms*

### *Catriona columbiana*

*Catriona columbiana* feeds on the athecate hydrozoans *Tubularia* sp. Although the feeding process itself has not been observed in situ*,* the underwater observations indicate that these molluscs feed on the soft part of the hydranth.

**Fig. 8:**
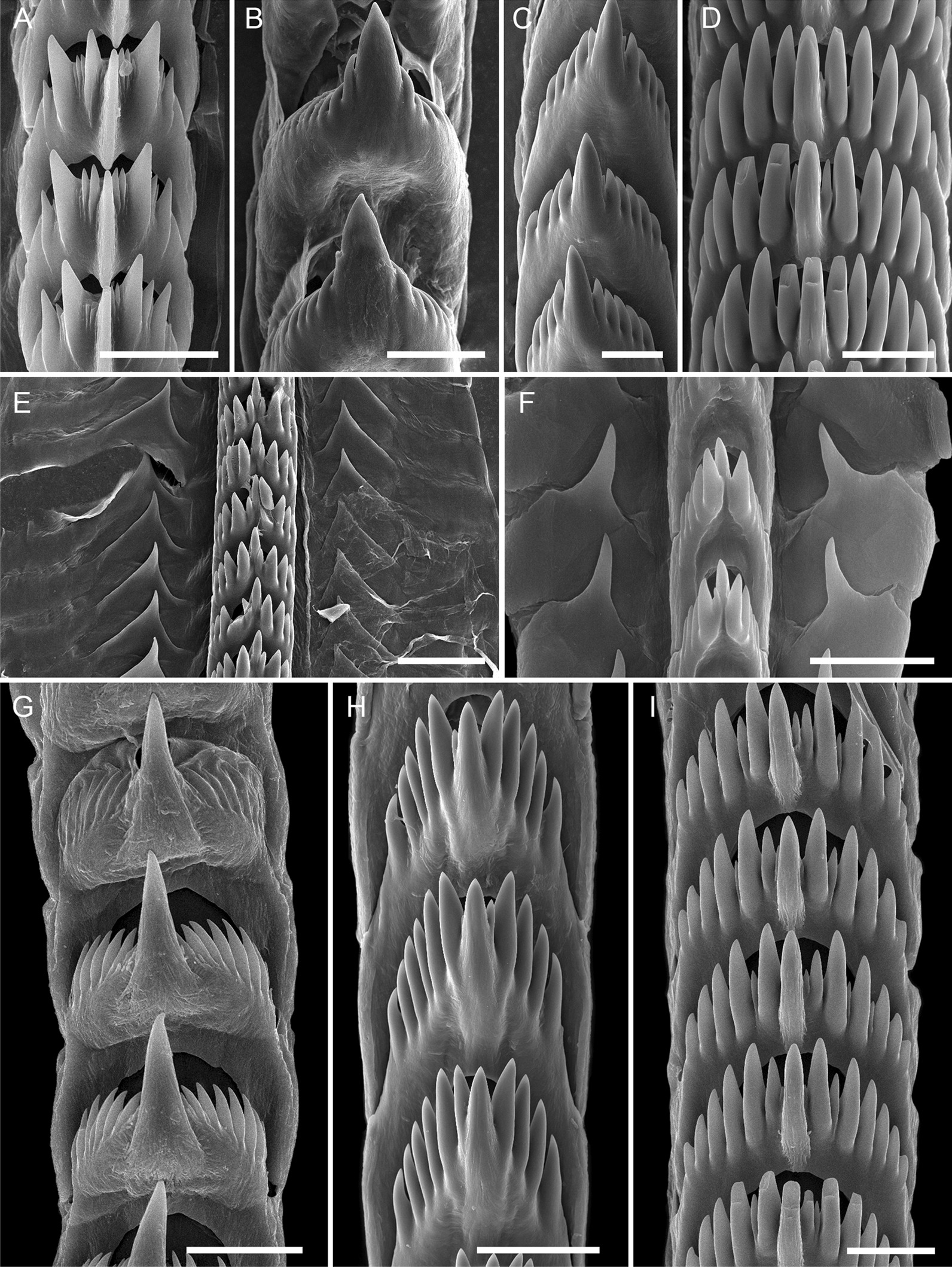
Radular morphology in different Fionidae species (SEM). **A** *Catriona columbiana.* **B** *Cuthona nana.* **C** *Cuthonella hiemalis.* **D** *Diaphoreolis viridis.* **E** *Eubranchus odhneri*. **F** *Eubranchus rupium.* **G** *Tergipes tergipes.* **H** *Zelentia pustulata.* **I** *Trinchesia ornata*. Scale bar: **A**, **C**, **D**, **F** 30 µm, **B** 40 µm, **E** 50 µm, **G**–**I** 20 µm

The jaws are thin with poorly developed masticatory processes and bristle-like denticles along the edge. The jaws do not take much part in the feeding process and have a primarily supportive function. The radula is uniserial, and the central cusp of the rachidian tooth is well-developed and narrow (Fig. 8A). Also, there are two or three lateral denticles on each side similar in size to the central cusp. Several additional small denticles are located between the central cusp and lateral denticles. Highly denticulated teeth are common for aeolids feeding on soft polyps. The radula of *Catriona columbiana* likely represents an adaptation for biting off the soft parts of polyps.

#### *Cuthona nana*

**Fig. 9:**
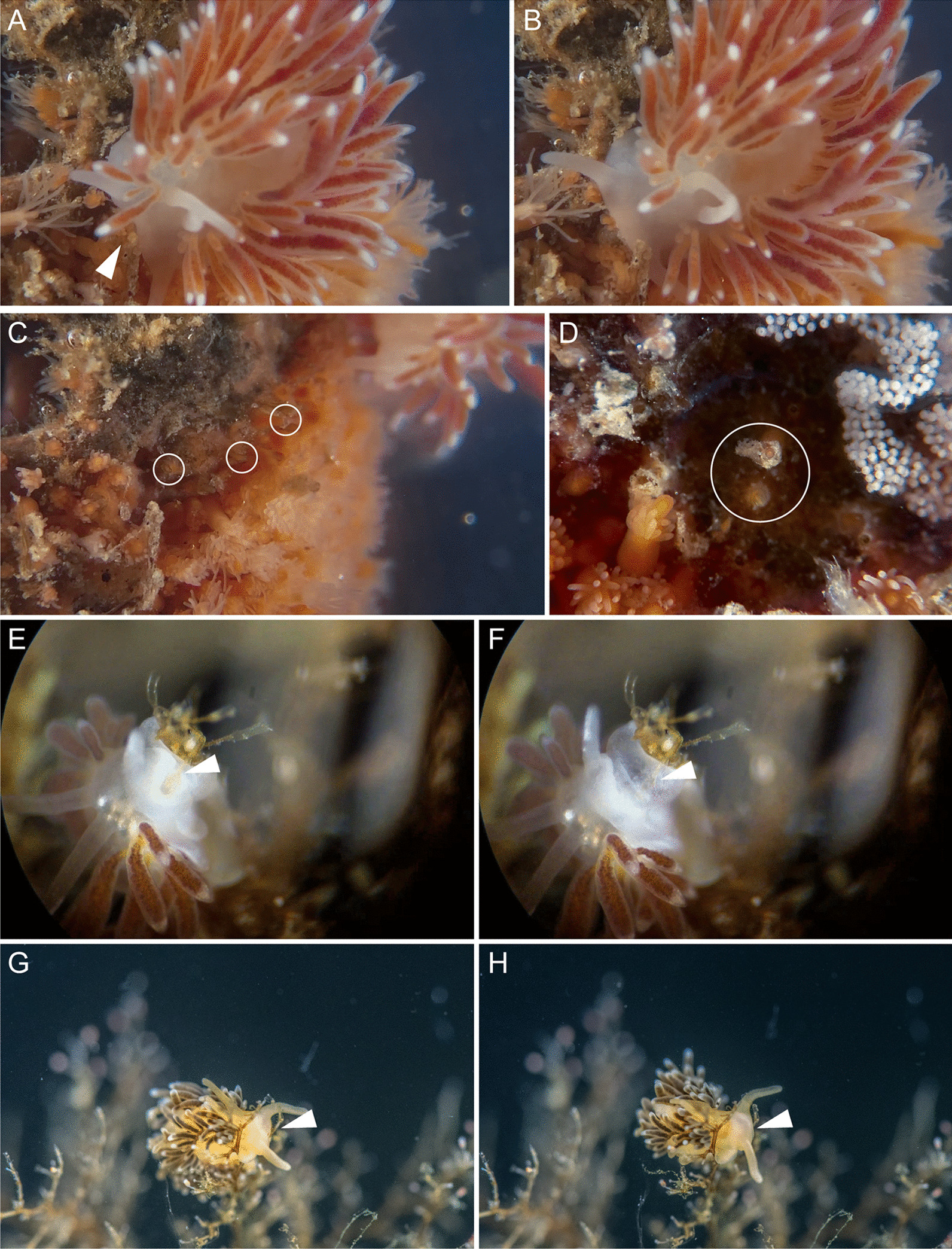
Feeding processes of several different Fionidae species. **A** *Cuthona nana*, discovering prey. **B** *Cuthona nana*, consuming prey. **C**, **D** *Hydractinia echinata* colony after *C. nana* feeding. Circles indicate stalks remaining after polyps consumed. **E** *Cuthonella concinna*, discovering prey. **F** *Cuthonella concinna*, end of feeding process, the hydrotheca of prey polyp is empty. **G** *Cuthonella hiemalis*, discovering prey. **H** *Cuthonella hiemalis*, consuming prey polyp. White arrowheads indicate prey polyp

*Cuthona nana* feeds on a small hydrozoan species *Hydractinia* sp., which grow on the shells of hermit crabs. Nudibranchs attack hydranths directly from above and consume the whole polyp, leaving only part of the stolon (Fig. 9A–D).

The jaw plates of *C*. *nana* are large with well-developed masticatory processes, bearing one row of scarce blunt-tipped conical denticles along the edge. The jaws are likely adapted for fixing the buccal complex on the feeding site. The radula of *C*. *nana* is uniserial (Fig. 8B). The central cusp is well-developed, twice longer and thrice broader than the lateral denticles, and it protrudes from the surface of the rachidian tooth.

#### *Cuthonella concinna*

*Cuthonella concinna* feeds on colonial hydroids of the family Sertulariidae, mostly on *Hydrallmania falcata* (Linnaeus, 1758). The mollusc finds a feeding spot on the hydrozoan colony using its outer lip and oral tentacles, and extends the buccal complex (Fig. 9E, F). It quickly pierces the perisarc of the colony with the radula and sucks in (or grabs, with the help of the radula) the hydranth tissue through the hole. *C. concinna* can consume one hydranth in 5–10 s. The specimen leaves irregularly shaped apertures on the perisarc. That indicates that *C. concinna* does not drill the perisarc but pierces it.

The jaws of *C. concinna* are large plates with well-developed masticatory processes bearing a row of conical denticles. The specimen probably holds the hydrozoan stolon using masticatory processes while feeding. The radula of *C. concinna* is uniserial. The central cusp is larger and longer than the outer denticles and the tooth edge forms a U-shaped cutting line. This tooth is likely suitable for both piercing (large protruding central cusp) and grabbing soft tissues (well-developed denticles, U-shaped form).

#### *Cuthonella hiemalis*, *C. osyoro*

The feeding mechanism of *Cuthonella hiemalis* is similar to that in *C. concinna*, except that this species feeds on hydrozoans of the family Campanulariidae (e.g., *Obelia longissima* (Pallas, 1766)) (Fig. 9G, H). The jaws and radular morphology in this species are similar to those in *C. concinna* (Fig. 8C). *Cuthonella osyoro* is commonly found on the same hydrozoans (Sertulariidae, Campanulariidae) and has similar radular morphology to other *Cuthonella* species; we therefore suggest that the feeding mode of this species is the same as in *C. concinna* and *C. hiemalis.*

#### *Diaphoreolis viridis*

**Fig. 10:**
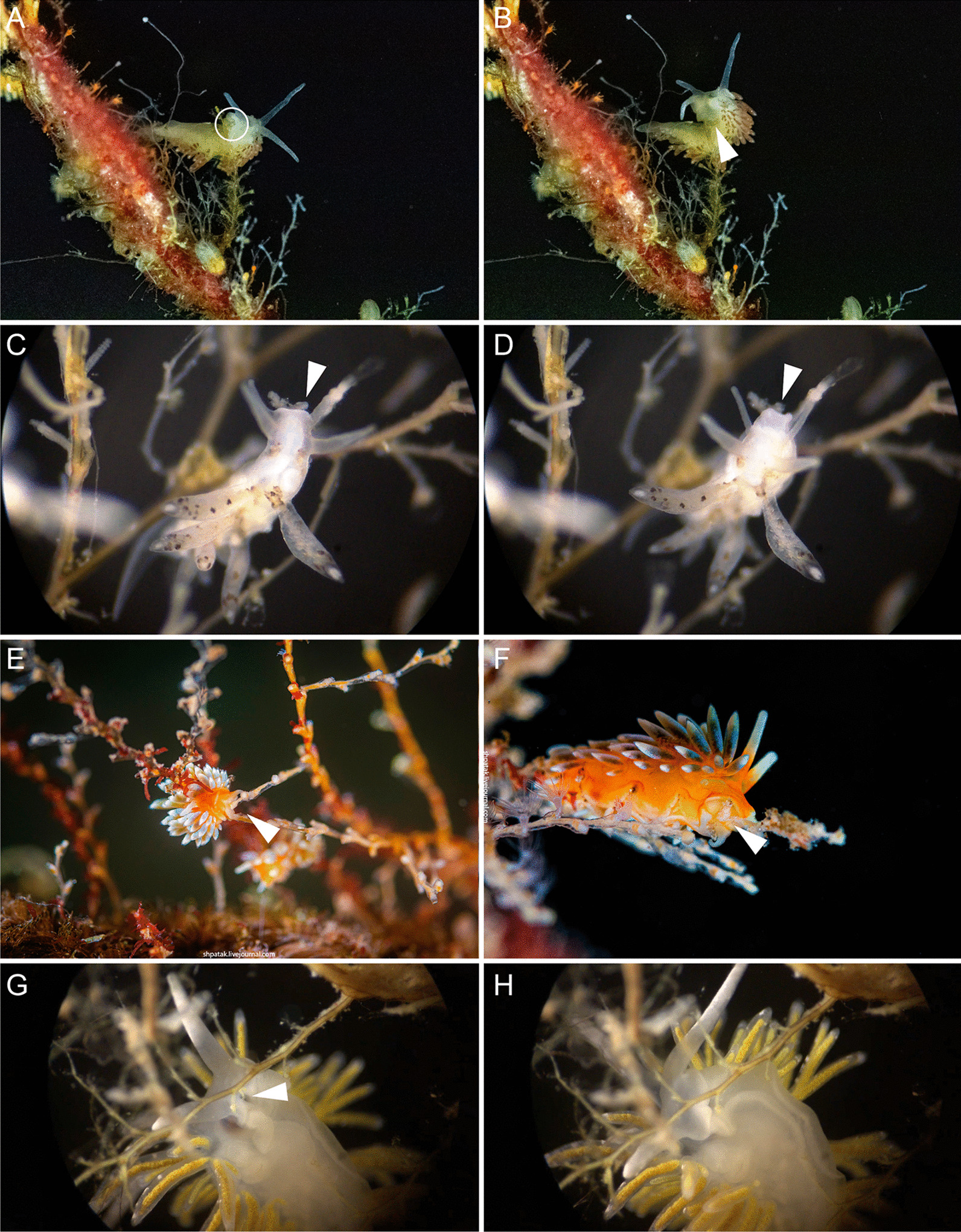
Feeding processes of several different Fionidae species. **A** *Diaphoreolis viridis*, discovering prey polyp, indicated by white circle. **B** *Diaphoreolis viridis*, end of the feeding process, the prey polyp hydrotheca is empty. **C** *Tergipes tergipes*, discovering prey polyp bud. **D** *Tergipes tergipes,* consuming prey polyp bud. **E** *Trinchesia ornata*, discovering prey polyp. Photo by A. Shpatak. **F** *Trinchesia ornata*, consuming prey polyp. Photo by A. Shpatak. **G** *Zelentia pustulata*, swallowing branch of *Halecium* sp. colony. **H** *Zelentia pustulata*, end of feeding process, branch of the *Halecium* sp. colony is fully consumed. White arrowheads indicate prey

*Diaphoreolis viridis* feeds on sertulariid hydrozoans *Diphasia fallax, Hydrallmania falcata,* and *Sertularia mirabilis*, but also on the smaller hydrozoans *Lafoea dumosa*, which overgrow sertulariid colonies. We observed the feeding behavior of *D. viridis* on *L. dumosa*. The hydrotheca of this cnidarian species lacks an operculum, allowing these molluscs to attack their prey from above and grab them using the radula (Fig. 10A, B).

The jaws of *D. viridis* are large plates with poorly developed masticatory processes. On the masticatory edge is one row of scarce blunt-tipped denticles. Most likely, the jaws are poorly adapted for biting off pieces of the prey or holding the buccal complex on the feeding site. Presumably, the jaws of *D. viridis* serve as the attachment site for the buccal muscles. The radula is uniserial and the teeth are comb-shaped, resembling those in *Aeolidia papillosa* (Fig. 8D).

#### *Eubranchus rupium*

A detailed description of the feeding mechanism of *Eubranchus rupium* was given previously by Mikhlina et al. [51]. *Eubranchus rupium* is a mechanical driller, boring holes in the perisarc of *Obelia longissima* and sucking hydrozoan tissue. It has a triserial radula with plate-like laterals adapted for mechanical drilling (Fig. 8F; [51]).

#### *Eubranchus odhneri*, *E. malakhovi*, and *E. pallidus*

The representatives of these species most probably feed on the colonial hydroids of the families Campanulariidae and Sertulariidae. Although we have not observed their feeding in situ and in vivo, the morphology of their radulae and jaws is similar to that of *E*. *rupium* (Fig. 8E). *Eubranchus odhneri* in the White Sea is often found on *Sertulariella gigantea,* and *E. malakhovi* occurs on different sertulariid hydrozoans [53]. At the same time, *E. pallidus* occurs in the same community as *E. rupium* in the Barents Sea. Taking into consideration the similarities in buccal armature across *Eubranchus* species, we suspect the feeding mechanism is also similar to the one in *E*. *rupium*.

#### *Tergipes tergipes*

*Tergipes tergipes* feeds on hydrozoan colonies of the family Campanulariidae (e.g., *Laomedea flexuosa* Alder, 1857, *Obelia longissima* (Pallas, 1766) or *Obelia geniculata* (Linnaeus, 1758)). The specimens attack polyp buds (Fig. 10C, D). A specimen holds the bud with its lips and the masticatory processes of its jaws, and grinds the bud using its radula. Sometimes the specimens attack polyps near the upper edge of their hydrothecae, but the polyps do not look damaged. Probably, these attacks are either unsuccessful or the nudibranch specimen bites off several tentacles.

The jaws of *T. Tergipes* are large, thin jaw plates with well-developed masticatory processes bearing a row of blunt-tipped, conical denticles. Most likely, the specimen uses the masticatory processes to hold the prey during the feeding process. The radula is uniserial, with a large central cusp protruding from the plane of the tooth (Fig. 8G). The central cusp is much larger than the lateral denticles. The specimen most likely uses the radula to bite off pieces of the polyp buds.

#### *Trinchesia ornata*

*Trinchesia ornata* is usually found on the sertulariid colonies (Fig. 10E, F). The molluscs attack the hydranth from above, enclosing the prey with the outer lip. The morphology of the radula and jaws is similar to that in *Diaphoreolis viridis* (Fig. 8I). The feeding mechanism is most likely similar in these species.

#### *Zelentia pustulata*

In the White and Barents Seas, *Zelentia pustulata* feeds on colonies of the hydrozoan *Halecium* sp. (Fig. 10G, H). The molluscs prefer the youngest branches with the thinnest perisarc. *Zelentia pustulata* swallows parts of the colony, starting from the branch tip. The mollusc then closes its jaws, and its masticatory processes cut off the branch.

The jaws of *Z*. *pustulata* are well-developed, and the masticatory process bears one row of sharpened conical denticles with secondary dentitions. Likely, the jaws serve for cutting the perisarc. The radula is uniserial, and the teeth are narrow and possess small conical central cusp and large denticles (Fig. 8H); this likely indicates the radula is used to grind food within the buccal cavity.

### Phylogenetic relationships within the family Fionidae *s.l.* and phylogenetic value of studied characters

**Fig. 11:**
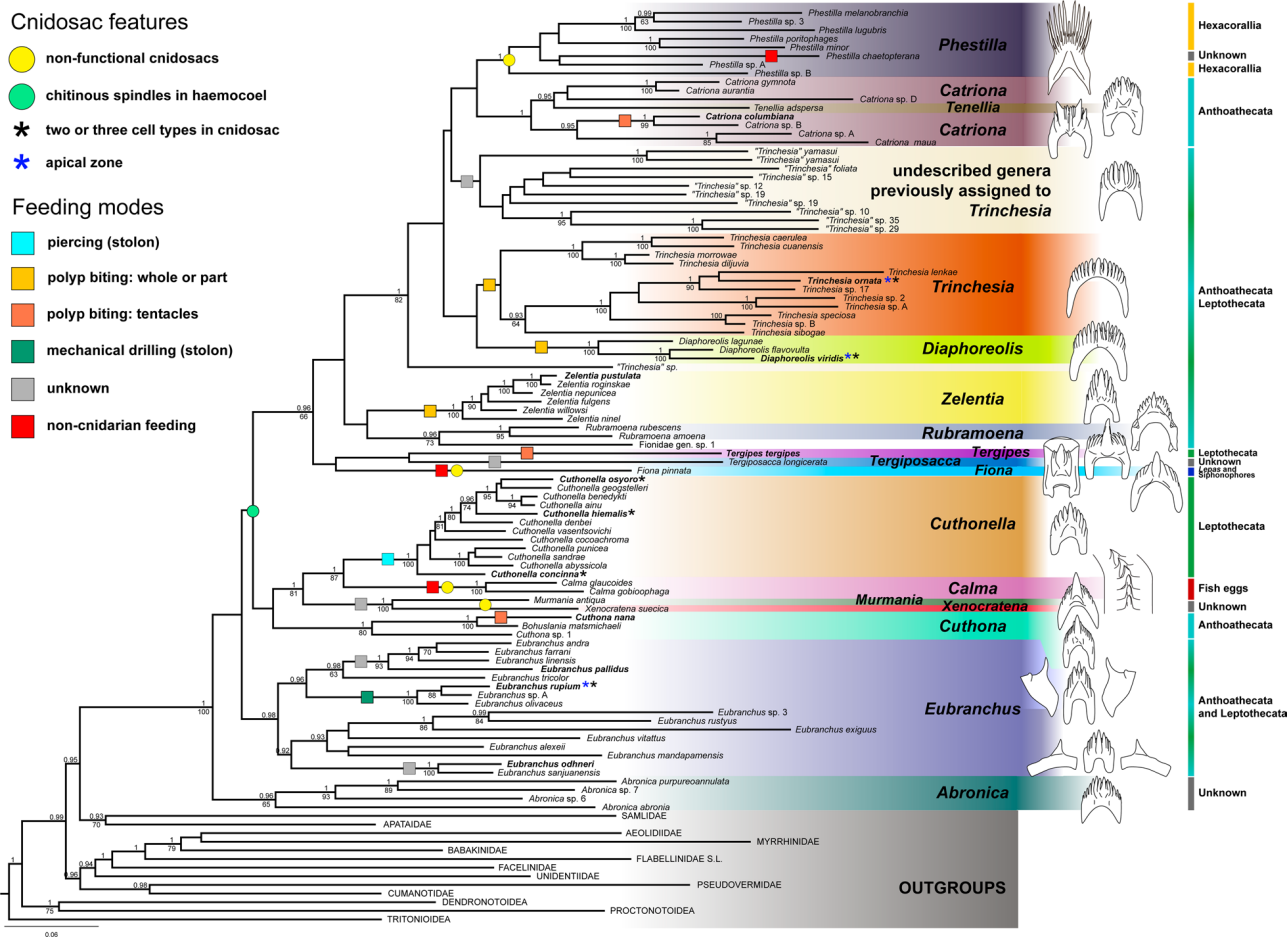
Maximum likelihood phylogenetic tree of the family Fionidae *s.l.* based on the concatenated dataset of three molecular markers (COI, 16S, H3). Species-level clades and outgroups are collapsed to a single branch. In four cases, two or three distinct species were collapsed to a single branch due to non-monophyly of its representatives (*Cuthona nana/divae, Cuthonella osyoro/soboli, Eubranchus viriola/andra, Eubranchus odhneri/malakhovi*). Numbers above branches indicate posterior probabilities from Bayesian Inference, numbers below branches—bootstrap support from Maximum Likelihood. Species studied in this work are highlighted in bold font. Several cnidosac features are mapped on respective branches or species as colored circles or stars, the feeding mechanisms of molluscs are mapped on respective branches as squares. For each genus the radular morphology and feeding objects (at high taxonomic level) are indicated

In our reconstruction, Fionidae *s.l.* represents a monophyletic group (Fig. 11, Additional files 5, 6: Data S1, S2), which is strongly supported by both the Bayesian inference (BI) and Maximum Likelihood (ML) analyses (PP (posterior probability from BI) = 1; BS (bootstrap support from ML) = 100). Most genera within the family are recovered as monophyletic and highly supported. The only exceptions are the genus *Catriona,* which is paraphyletic because *Tenellia adspersa* is positioned within it (PP = 1), and the genus *Cuthona,* containing *Bohuslania matsmichaeli,* which represents sister relationships to *Cuthona nana* (PP = 1; BS = 100). The deep relationships within the family are poorly supported. However, our analysis supports the monophyly of a clade uniting *Diaphoreolis, Trinchesia, Catriona, Tenellia* and *Phestilla* (PP = 1; BS = 82) and a clade formed by the genera *Cuthonella, Calma, Murmania* and *Xenocratena* (PP = 1; BS = 81).

## Discussion

### Diversity of cnidosac fine structure within Fionidae *s.l.*

Our results indicate that the cnidosacs of different Fionidae *s.l. *species fit the common aeolid features: there is a single cnidosac per ceras, connected to the digestive gland diverticulum by a narrow channel (Figs. 1, 2, 3). The cnidosac contains three zones of different function (the proliferation zone, the cnidophage zone and the cnidopore). Cnidophages contain different types of NCs with the types depending on diet (Additional file 3: Table S3). The discharging of the cnidosac occurs with the help of musculature contraction and the injection of cnidophages with NCs in the water. Although we did not detect any significant divergence from the general scheme, several fine morphological features of the cnidosac vary greatly across the studied fionid genera (Fig. 1, Additional files 7–15: Fig. S1–S9). Musculature layers surrounding the cnidosac vary in form and degree of development among species. In several species, like *Catriona columbiana* and *Zelentia pustulata,* the cnidosac is thin-walled and the layers of circular and longitudinal musculature are hardly distinguishable (Fig. 2D, F). In other species like *Cuthona nana* and *Diaphoreolis viridis,* these layers are well-developed and form a thick mesh consisting of a mixture of differently oriented filaments (Fig. 2C, E). This agrees with data from previous studies [20, 26], though we did not find any correlation between the density of the muscular layer and the type of sequestered NCs or any ecological traits that could explain this variation. Goodheart et al. [20] suggested that musculature thickness may correlate with predator pressure, but it seems unlikely as species occurring sympatrically on the same hydrozoans and possessing cryptic external morphology (*e.g. Eubranchus rupium* and *Tergipes tergipes*), have different musculature layer thicknesses. Variation of the musculature thickness may be explained by the differences in the developmental stages of studied specimens, as was shown recently for *Berghia stephanieae* [66]. However, all molluscs studied here were fully mature (specimens of each species commonly copulated with each other) to avoid possible ontogenetic variation.

One of the most variable features is the NC arrangement within the cnidophages (Figs. 1, 4). For example, in some species like *Tergipes tergipes, Diaphoreolis viridis,* and *Eubranchus odhneri*, the location of NCs is not ordered within the cell, and they are enclosed in large vacuoles (Fig. 3D, I; 4D, E; Additional file 10: Fig. S4D). Other species demonstrate ‘closer’ relationships between NCs and the cnidophage membrane, meaning they are arranged very close to the cnidophage membrane at the apical end like in species *Eubranchus rupium, E. pallidus,* and *Trinchesia ornata* (Fig. 4A, Additional files 11, 14: Fig. S5C, S8B, C). In *Cuthona nana* and *Catriona columbiana,* NCs are arranged in a circle around the nucleus and most cell organelles; moreover, in the latter case NCs are oriented by a cap to the cell membrane (Fig. 4F, Additional files 7, 8: Fig. S1D, G, S2D)*.* This feature is particularly interesting as it resembles the arrangement of NCs in cnidarian nematocytes (cells containing NCs in Cnidaria). It is possible that NCs in this position are exploded more effectively during cnidosac discharging as cnidophage damage is likely unnecessary for NCs to fire (unlike cnidophages with NCs in the central part of the cytoplasm). Finally, in *Zelentia pustulata* the NCs are closely adjacent to the lumen, which is underlined by numerous large vacuoles located in the basal parts of cnidophages adjacent to the cnidosac muscular wall (Fig. 4C). In most cases we did not detect the same phagosome membrane as is present in *Tergipes tergipes* and *Diaphoreolis viridis.* NC arrangement is likely not dependent on the NC type as well. At least *D. viridis, Z. pustulata, Trinchesia ornata,* and species of the genus *Eubranchus* sequester similar NCs (euryteles, mastigophores and isorhizas), but their position within cnidophages is different, as explained above. It should be also noted that these different arrangements do not correlate to the recovered phylogenetic relationships (Fig. 11), and it is not possible to give any ecological explanation as our morphological and ecological data are limited compared to the great biodiversity of Fionidae *s.l.*.

Another variable feature is the number of sequestered NCs: some cnidophages are small and contain few NCs, like in *Cuthonella hiemalis* and *E. rupium* (Figs. 4A, B; Additional file 9: Fig. S3, Additional file 12: Fig. S6), while in other species, cnidophages are very large cells with a dozen of NCs per cell, like in *E. odhneri* (Fig. 3D, I, see also [53]). In *Tergipes tergipes,* cnidophages are also large and contain numerous NCs, but many more intact NCs are located in the cnidosac lumen (Additional file 14: Fig. S7). Commonly, the number of NCs in the cnidosac correlates with the number of cell types in the cnidophage zone (*e.g.,* cnidosacs with few NCs contain additional cell types except cnidophages) (see below). It may be assumed that animal or cnidosac size may show a correlation with NC number within cnidophages. However, we did not find any correlation in this case, as the cnidosacs of *Cuthonella hiemalis, Diaphoreolis viridis, Zelentia pustulata,* and *Eubranchus odhneri* are of about same size (Figs. 2B, D, E, 3I), but the NC number is different. Another explanation may be the time when the animals last fed, though all studied material was collected with their food, and relaxed and fixed following the same protocol with the same exposition time; also, the morphology of specimens collected at different times and sometimes in different years was found to be similar, so we do not assume any variation in this case.

Although the cnidosac lumen may also vary greatly among the different fionid species, the significance of this variation is not clear. The lumen is commonly very large in species with large, voluminous cnidophages like *Tergipes tergipes* and *Zelentia pustulata* (Additional files 13, 15: Figs. S7, S9). Species with few NCs and additional cell types usually have a very small lumen, like in *Eubranchus rupium* and *Cuthonella hiemalis* (Additional files 9, 12: Figs. S3, S6). This may imply that a large, voluminous lumen is the characteristic trait for species with many NCs, as an enlarged internal space eases NC processing. This hypothesis is indirectly supported by data on the cnidosac structure in species that have lost the ability to consume functional NCs, but which still have a cnidosac with vacuolized cnidophages (*i.e., Phyllodesmium,* see for example Supplementary files in [20]): in this case the lumen is absent. However, this issue clearly requires additional study, as there are other examples of cnidosacs with many nematocysts but an extremely small lumen (*i.e., Catriona columbiana*, Additional file 7: Fig. S1).

### Additional cell types in cnidosacs

The unexpected diversity of cnidosac structure within Fionidae *s.l.* is linked with the diversity of cell types within its epithelial layer. Most of the studied species have only cnidophages as the main cell type in the cnidosac. However, in *Eubranchus rupium* and *Cuthonella hiemalis,* we detected cells that surround cnidophages and do not contain NCs (Fig. 5C, D). Possibly these cells are cnidophages that were not able to consume NCs due to the small number of NCs left after feeding. Interstitial cells were detected in *Aeolidia papillosa *[24], and they are likely present in other Aeolidiidae. These cells in *A. papillosa* surround cnidophages and do not contain NCs (as those in *E. rupium* and *C. hiemalis*); instead, they possess granular chitin and, probably, act as supportive and protective cells. It is not clear whether cells without NCs in fionids are homologous to interstitial cells in *A. papillosa.* However, considering the high number of these cells in the upper parts of the cnidosac in *Eubranchus rupium* and *Cuthonella hiemalis,* we provisionally designate them as interstitial cells until shown otherwise.

In the case of *Cuthonella hiemalis,* we also detected specific cells with granular electron-dense inclusions in numerous vacuoles (Fig. 5D). These cells also occur in the digestive gland diverticula and appear in other *Cuthonella* species as well (Additional file 9: Fig. S3). The function of these cells and nature of electron-dense granular compounds is not known, but their occurrence in both cnidosac and the digestive gland diverticulum may indicate their relation to the metabolic processes occurring throughout the digestive system.

In most studied fionids, the cnidopore area is simple (Figs. 2, 3): it is adjacent to the epidermis. In some cases, we detected the close contact of epidermis and cnidosac epithelium, like in *E. pallidus* and *Trinchesia ornata* (Additional files 11, 14: Figs. S5G, S8G). The cnidosac epithelial layer in the cnidopore zone consists of either normal cnidophages (for most of the species studied), or of undamaged interstitial cells (for *Eubranchus rupium* and *Cuthonella*) (Additional file 8: Fig. S2B). In the case of *Cuthona nana, Catriona columbiana,* and *Trinchesia ornata,* we detected a visible invagination of the epidermis in the cnidopore (Fig. 3E, F, Additional file 14: Fig. S8G). At the same time, *Diaphoreolis viridis* and *Trinchesia ornata* have a prominent cnidopore area forming a narrow channel lined by cells with signs of degradation, *e.g.* distorted, lobe-shaped nucleus and foamy cytoplasm (Fig. 5A, B). Possibly these cells are discharged, damaged cnidophages that have expelled NCs.

### Unique cell types in haemocoel

Our results confirm reports of the presence of *cellules speciale* in fionid species [26, 67–70]. These enigmatic cells are located in the haemocoel near the digestive gland and the cnidosac; each cell has a very granulated cytoplasm and a large nucleus, and shows positive nuclear staining (Figs. 2, 6A–C). They were suggested to be storage cells [26] or to play a role in protein metabolism [70]. Edmunds [26] suggested a storage function for these cells due to their relation to the digestive gland diverticula and their increased number following feeding [36, 37]. Schmeckel [70] was the first to study the ultrastructure of these cells and concluded that it is unlikely that they function as storage, as no storage vacuoles are found within these cells. The high density and amount of the granular endoplasmic reticulum (Fig. 5C) suggest these cells have high synthetic activity, likely related with the haemocoel protein metabolism [70]. For now, *cellules speciale* were found in different Fionidae* s.l.* species [26, this study], and several representatives of Aeolidiidae, Facelinidae, and Myrrhinidae [26, 71]. Proteins produced by the granular endoplasmic reticulum may be identified in further studies of haemocoel transcripts.

A distinctive feature for all Fionidae* s.l.* studied except representatives of the genus *Eubranchus* is the presence of haemocoel cells that contain vacuoles with chitinous spindles. These cells are commonly detected with Calcofluor White staining for amorphic chitin using CLSM (Fig. 2, *hc*), but also appear on TEM sections (Fig. 5D). Since the chitinous elements are characteristic in the cladobranch epidermis (Figs. 2, 3, white color), it may be suggested that these cells are subepidermal. However, we did not find any viable connection through a channel, or any signs that these cells have epidermal origin. Storage and use of chitinous elements in the nudibranch epithelium are believed to be a protective mechanism against NC discharging [72], but the function of these cells in haemocoel is unclear, as haemocoel does not come into contact with intact NCs. It should also be noted that cells with chitinous spindles are commonly found adjacent to *cellules speciale* (Figs. 2E, 5D), indicating they may have associated activity.

## Correlation of cnidosac morphology and diet preferences

According to our results, the diversity in cnidosac fine structure within Fionidae correlates with the diversity of radular morphology and the feeding preferences of each species (Figs. 1, 11). It was suggested previously that types of obtained NCs may determine the fine features of cnidosacs, like in representatives of the family Aeolidiidae [20, 23, 24]. Species in this group are specialized on anemones and some other hexacorals and sequester extremely long and narrow mastigophores. As a result, their cnidosacs contain specific interstitial cells with numerous chitinous spindles; these cells surround cnidophages and line the cnidopore channel, possibly for additional protection from kleptocnides [24]. These cells were found in at least *Aeolidia papillosa* [23]; however complex cnidopores of similar morphology are found in other Aeolidiidae [20], so they may be formed by the same interstitial cells. In the case of the representatives of the family Fionidae *s.l.,* we did not find any clear correlation between consumed NC types and specific cnidosac features. Instead, species with similar NC types and diet (in example, *Tergipes tergipes* and *Eubranchus rupium*) show great differences in cnidosac ultrastructure (Figs. 1, Additional files 12, 13: Figs. S6, S7), which may be explained by other ecological properties, *e.g.,* the feeding mode of the mollusc. For instance, both species feed on *Obelia longissima,* but *T. tergipes* has a high number of kleptocnides in the lumen and cnidophages, and *E. rupium* has only a few NCs in the cnidophages as well as NC-free interstitial cells (Additional file 12: Fig. S6). This could be because *T. tergipes* feeds upon the hydroid’s tentacles and hydranths, which contain high concentrations of NCs, whereas *E. rupium* avoids the polyp buds and feeds directly on the hydrozoan internodes, which contain only a few mature or pre-mature NCs. The same is characteristic for representatives of the genus *Cuthonella,* which pierce the hydrozoan perisarc and grab soft tissues (Fig. 8E, F); their cnidosacs also contain a relatively low number of NCs and three distinct cell types (Additional file 9: Fig. S3). *Diaphoreolis viridis* and *Trinchesia ornata* demonstrate several similarities in cnidosac structure, with highly vacuolated cnidophages and a distinct cnidopore area lined with degraded cells (Additional files 10, 14: Figs. S4, S8) in combination with similar radular morphology (Fig. 8D, I) and a presumable feeding mode (Fig. 10A, B, E, F).

Another notable correlation is between cnidosac ultrastructural complexity and prey repertoire. Narrowly specialized nudibranchs tend to have cnidosacs that are more diverged from the generalized model. *Cuthona nana, Catriona columbiana,* and *Zelentia pustulata*, who feed on a specific hydroid species (*Hydractinia* sp.*, Tubularia* sp. and *Halecium* sp. respectively) have specific patterns of NCs positioning within cnidophages. In *Cuthona nana,* NCs are organized in a circle around the nucleus (Fig. 3E), in *Catriona columbiana* NCs are oriented by cap to the cell membrane (Fig. 4F, Additional file 7: Fig. S1D, G, H), and in *Z. pustulata* NCs form a dense layer in the apical parts of cnidophages adjacent to the lumen (Fig. 4C, Additional file 15: Fig. S9E, F). At the same time, *Diaphoreolis viridis* has wider prey preferences and is a better fit for the general plan of cnidosac morphology found in other aeolid species (*i.e.* some representatives of the families Flabellinidae, Facelinidae, see for example data in Goodheart et al. [20]) as it has more or less irregularly placed NCs of different types located in phagosomes.

It should be also noted that studies of ecological traits are extremely challenging. Associating nudibranchs with cnidarian species is commonly interpreted as a trophic connection between them. However, it actually requires further investigation as nudibranchs may feed on smaller hydrozoans overgrowing the host species [31], like in the case of *Diaphoreolis viridis* and *Lafoea dumosa* (Fig. 10A, B). Also, it is not easy to determine the exact feeding mode, as it requires comprehensive in situ and in vivo observations. In most species, it remains poorly studied, hindering our understanding of the precise ecological characteristics of most groups.

### Correlation of dietary preferences, radular morphology, and phylogenetic relationships within Fionidae

A combination of feeding mode and prey species determines nudibranch radular morphology [17, 50, 51, present data]. In Fionidae *s.l.,* many groups have evolved a certain feeding mechanism or feed on a specific prey species. As a result, each genus is characterized by specific radular morphology (Fig. 11). All *Eubranchus* have a triserial radula with plate-like lateral teeth, and all known intrageneric variation relates to the form of the lateral teeth and number of denticles on the rachidian tooth (Fig. 8E, F), see also [51, 73]. In *Cuthona, Tergipes, Tergiposacca, Fiona, Murmania,* and *Xenocratena* the central cusp is much larger than the lateral denticles (Fig. 8B, G), but the form of the tooth is different [40, 41, 45, this study]. The central cusp is reduced in *Catriona* and *Phestilla,* while the lateral denticles are enlarged in a different pattern (Figs. 8A, 11). The central cusp and the lateral denticles are almost similar in size in *Tenellia, Zelentia, Cuthonella, Rubramoena,* and some of the former “*Trinchesia”* (*i.e., “Trinchesia” yamasui*), but the tooth form is different in each genus (Fig. 8B, G), see also [40, 44]. Phylogenetically close genera *Diaphoreolis* and *Trinchesia* display similar morphology: a wide rachidian tooth with many sharp denticles of same size (Fig. 8D, I) resembling very wide teeth with numerous blade-like denticles found in Aeolidiidae [74]. *Calma* has an unusual radular ribbon fused in a single plate with only small dentition on the working plane (Fig. 11), see [48]. The high phylogenetic signal of radular characters was previously indicated [44], and although some similarities in radular morphology occur across genera, we find the specific radular characters correlate with the prey species and/or represent their feeding mechanisms. As seen from the Fig. 11, the shift to the exclusively non-cnidarian prey occurs at least twice in genera *Fiona* and *Calma.* Among cnidarian-feeding fionids, some specialization is also obvious: *Phestilla* feeds on scleractinian corals [18], *Cuthonella* prefers leptothecate hydrozoans, not closely related *Catriona* and *Cuthona* feed on different Anthoathecata, and only within *Eubranchus* and former “*Trinchesia”* genera (*Trinchesia, Diaphoreolis, Zelentia, Rubramoena*) is the large-scale shift between different hydrozoan taxa common. Although our study is limited in the number of studied species, it is obvious that Fionidae *s.l.* represents a high diversity in prey species and feeding mechanisms, which is likely a result of prey-specific adaptive radiation.

### Evolutionary implications of nematocyst sequestration within Fionidae

The loss of functional cnidosacs occurred at least three times within Fionidae* s.l.*, in the case of the genera *Phestilla, Calma* and *Fiona*, which agrees with the analysis of Goodheart et al. [20]. Goodheart et al. [20] suggested functional cnidosacs are also lost in representatives of the genus *Tergipes* (in *Tergipes tergipes* and *T. antarcticus*) [75]*,* however, in the present work we show that the cnidosac of *Tergipes tergipes* is well-developed and contains functional NCs (Additional file 13: Fig. S7). This suggests that cnidosac loss due to shift in diet may occur even within a single genus. At the same time, the position of *Tergipes antarcticus* on the Fionidae tree remains questionable: a single attempt to incorporate this species into the broad phylogeny showed its unstable position on the phylogenetic tree [40]. In the analysis of Goodheart et al. [20] the lower taxon sampling in contrast to [40], cannot undoubtedly support the monophyly of clade uniting *T. antarcticus* and *T. tergipes,* thus suggesting *T. antarcticus* may represent a separate phylogenetic entity.

The absence of a functional cnidosac is a specific trait of the genera *Calma, Fiona* and *Phestilla* [20]*.* The loss of the ability for NC sequestration clearly relates to a shift either to a non-cnidarian food source or to a cnidarian species without certain NC types. For instance, representatives of the genus *Calma* feed on fish eggs [47], and the *Fiona* prey spectrum commonly includes stalked barnacles [49, 76]. Within the corallivorous genus *Phestilla,* one species *P. chaetopterana* also shows no association with cnidarian prey, exhibiting instead a symbiotic association with the annelid *Chaetopterus* [77]. Other *Phestilla* feed on scleractinian corals [18], which would imply a presence of functional cnidosacs. However, most of the *Phestilla* feed on the corals containing only spirocysts and no nematocysts [78], which limits the ability of nudibranchs to sequester and use these cnidae. A single exception is *Phestilla melanobrachia* which feeds on *Tubastrea,* for which the presence of holotrichous isorhizas, mastigophores, and amastigophores was shown [79]. Nevertheless, *P. melanobranchia* lacks functional cnidosacs as well [20]. The reconstruction of ancestral states (diet) within *Phestilla* [18] implies the ancestral host species for this group is *Porites,* and the shift to feeding on *Tubastrea* was likely a secondary one. Previous studies suggested that *Phestilla* sequester secondary metabolites from their prey, which changes the defensive strategy from mechanical (NCs) to chemical [38], and in this case the secondary switchback to the prey species with proper NCs does not lead to the sequestration of functional kleptocnidae.

Although this study demonstrates that diversity of feeding mechanisms and prey species may have a certain phylogenetic signal, no such direct correlation can be found for cnidosac morphology. The cnidosac cells and NC assemblage, the fine morphology of different cnidosac zones, and the development of muscular layers may vary greatly among different genera or even within a single genus (e.g.,* Eubranchus*). The type of sequestered NC is undoubtedly dependent on the prey species, and may vary within a single genus (e.g.,* Cuthonella, Eubranchus*). In some cases, a minimal shift in the prey species (meaning a shift to another hydrozoan family without changing the feeding mechanism) results in a different set of sequestered NCs. In example, different *Cuthonella* species pierce the hydrozoan perisarc of different taxonomic groups: *Cuthonella hiemalis* feeds on the family Campanulariidae and sequesters isorhizas and mastigophores (Fig. 4B, Additional file 9: Fig. S3), while *Cuthonella concinna* is associated with the Sertulariidae species, thereby its cnidosac lacks isorhizas and has different forms of mastigophores (Fig. 3C). The greatest diversity of sequestered NCs was demonstrated for species with a variety of prey species (*Diaphoreolis viridis*). Also, some morphological characters, i.e., NCs number and arrangement, may be similar in species consuming similar part of prey as explained above (*i.e. Cuthonella hiemalis* and *Eubranchus rupium*). Vice versa, cnidosac morphology is different in species with similar prey species but different feeding mechanisms (i.e.,* Tergipes tergipes* and *Eubranchus rupium*). These data indicate that cnidosac morphology likely follows microevolutionary prey shifts, in other words switching between prey species and changing the prey site, and may be a useful indicator when studying the ecological features of particular species.

## Conclusions

The nudibranchs of the family Fionidae *s.l.* have a diverse feeding style and prey choice, especially considering genus-specific differences of buccal armature characters. While larger-scale prey shifts (i.e. shifts between cnidarian and non-cnidarian prey) rarely occur within the Fionidae *s.l.,* microevolutionary shifts between different hydrozoan species within a single genus are much more common. The diversity of radular morphology shows a correlation with dietary and feeding mechanism shifts, and represents a unique pattern for each of the large Fionidae groups. At the same time, the cnidosac morphology demonstrates considerable changes even when switching between similar hydrozoan species or changing the feeding site on the same prey species (*i.e.* feeding on hydrozoan buds vs feeding on internodes). The cnidosac morphology is therefore closely tied to the fine ecological characteristics of nudibranch species.

## Supplementary Information

**Additional file 1**. **Table S1**. Number of specimens of each species used in this study. Abbreviations: spec = specimens.

**Additional file 2.** **Table S2**. Specimens used for molecular analysis. Voucher numbers and GenBank accession numbers are given

**Additional file 3**. **Table S3**. Comparison of NCs types found in nudibranch cnidophages with NCs found in the corresponding prey species.

**Additional file 4**. **Table S4**. Cnidosac features, buccal armature morphology and prey species of studied nudibranch species.

**Additional file 5**. **Data S1**. Unedited maximum likelihood phylogenetic tree based on the concatenated dataset of three markers (COI+16S+H3) in NEWICK format.

**Additional file 6**. **Data S2**. Unedited Bayesian phylogenetic tree based on the concatenated dataset of three markers (COI+16S+H3) in NEWICK format.

**Additional file 7**. **Figure S1. Catriona columbiana*, *cnidosac morphology.** A—generalized scheme of cnidosac structure. B—cross-section through cnidophage zone. C—cnidophage zone. D—cnidophage cell membrane. E—NC wall. G—NCs in cnidophages. F—epidermis. H—NC within cnidophage. Abbreviations: cnph—cnidophage, chs—chitinous spindles, dg—digestive gland, ep—epithelium, glc—cells with granules, hc—cells with chitinous spindles, he—haemocoel, lu—lumen, mb—body musculature, mc—cnidosac musculature, nc—NCs, nu—nucleus, vc—vacuoles with chitinous spindles. Scale bars in µm.

**Additional file 8**. **Figure S2. Cuthona nana, cnidosac morphology.** A—generalized scheme of cnidosac structure. B—cnidopore zone, black arrowheads indicate basal laminae. C, F—epidermis. D—cnidophage. E—NCs within cnidophage. G—haemocoel with *cellules speciale *(cs). H—*cellules speciale*. Abbreviations: cnph—cnidophage, chs—chitinous spindles, cs—*cellules speciale*, dg—digestive gland, ep—epithelium, er—endoplasmic reticulum, hc—cells with chitinous spindles, he—haemocoel, lu—lumen, mb—body musculature, mc—cnidosac musculature, nu—nucleus, nc—NCs, smg—subepidemal mucus gland, va—vacuoles, vc—vacuoles with chitinous spindles. Scale bars in µm.

**Additional file 9**. **Figure S3. Cuthonella hiemalis*, *cnidosac morphology.** A—generalized scheme of cnidosac structure. B, I—cnidopore zone. C—epidermis. D, E—haemocoel. F, G—cnidophage zone. H—nematocysts (nc) within cnidophages. J, K—*cellules speciale*. Abbreviations: cnph—cnidophage, ci—cilia, chs—chitinous spindles, cs—*cellules speciale*, dg—digestive gland, ep—epithelium, er—endoplasmic reticulum, gc—cells with granular compound, gv—vesicles with electron-dense granules, hc—cells with chitinous spindles, he—haemocoel, ic—interstitial cells, lu—lumen, mb—body musculature, mc—cnidosac musculature, nu—nucleus, ncl—nucleolus, nc—NCs, va—vacuoles, vc—vacuoles with chitinous spindles. Scale bars in µm.

**Additional file 10**. **Figure S4. Diaphoreolis viridis*, *cnidosac morphology. **A—generalized scheme of cnidosac structure. B, C—apical zone. D, E—cnidophage zone. F, G—NCs within cnidophages. Abbreviations: cnph—cnidophage, cns—cnidosac, chs—chitinous spindles, cs—*cellules speciale*, dg—digestive gland, ep—epithelium, hc—cells with chitinous spindles, he—haemocoel, lu—lumen, mb—body musculature, mc—cnidosac musculature, nu—nucleus, nc—NCs, va—vacuoles, vc—vacuoles with chitinous spindles. Scale bars in µm.

**Additional file 11**. **Figure S5. Eubranchus pallidus, cnidosac morphology.** A—generalized scheme of cnidosac structure. B, C—cnidophage zone. D—cnidosac muscular wall. E—NCs within cnidophage. F—epidermis. G—cnidopore zone. Abbreviations: cnph—cnidophage, chs—chitinous spindles, cs—*cellules speciale*, dg—digestive gland, ep—epithelium, er—endoplasmic reticulum, ic—interstitial cells, he—haemocoel, lu—lumen, mb—body musculature, mc—cnidosac musculature, muc—mucous cell, n—nucleus, nc—NCs, ncl—nucleolus, vc—vacuoles with chitinous spindles. Scale bars in µm.

**Additional file 12**. **Figure S6. Eubranchus rupium*, *cnidosac morphology. **A—generalized scheme of cnidosac structure. B—cnidopore zone. C—epidermis. D—haemocoel. E—cnidosac entrance. F, G—cnidophage zone. H—NCs within cnidophage. Abbreviations: ci—cilia, cnph—cnidophage, chs—chitinous spindles, cs—*cellules speciale*, dg—digestive gland, ep—epithelium, er—endoplasmic reticulum, ic—interstitial cells, he—haemocoel, lu—lumen, mb—body musculature, mc—cnidosac musculature, mv—microvilli, muc—mucous cell, nu—nucleus, nc—NCs, ncl—nucleolus, va—vacuoles, vc—vacuoles with chitinous spindles, vn—vacuoles with NCs. Scale bars in µm.

**Additional file 13**. **Figure S7. Tergipes tergipes*, *cnidosac morphology.** A—generalized scheme of cnidosac structure. B—proliferation zone. C, D—cnidophage zone. E—cnidopore zone. F—epidermis. Abbreviations: apc—cell without NCs in cnidopore zone, cnph—cnidophage, cns—cnidosac, chs—chitinous spindles, cs—*cellules speciale*, dg—digestive gland, ep—epithelium, hc—cells with chitinous spindles, he—haemocoel, lu—lumen, mb—body musculature, mc—cnidosac musculature, n—nucleus, nc—NCs, ncl—nucleolus, va—vacuoles, vc—vacuoles with chitinous spindles. Scale bars in µm.

**Additional file 14**. **Figure S8. Trinchesia ornata*, *cnidosac morphology.** A—generalized scheme of cnidosac structure. B, C—cnidophage zone. D—cnidopore zone. E, F—NCs within cnidophage. H—epidermis. G—epidermal invagination in cnidopore area. Abbreviations: apc—cells without NCs in cnidopore zone, cnph—cnidophage, cns—cnidosac, chs—chitinous spindles, cs—*cellules speciale*, dg—digestive gland, ep—epithelium, er—endoplasmic reticulum, hc—cells with chitinous spindles, he—haemocoel, lu—lumen, mb—body musculature, mc—cnidosac musculature, mv—microvilli, n—nucleus, nc—NCs, va—vacuoles, vc—vacuoles with chitinous spindles. Scale bars in µm.

**Additional file 15**. **Figure S9. Zelentia pustulata*, *cnidosac morphology.** A—generalized scheme of cnidosac structure. B, C—epidermis. D—haemocoel. E—cnidophage zone, cross-section. F—cnidophage. G—NCs within cnidophages. Abbreviations: cnph—cnidophage, chs—chitinous spindles, cs—*cellules speciale*, dg—digestive gland, ep—epithelium, er—endoplasmic reticulum, hc—cells with chitinous spindles, he—haemocoel, lu—lumen, mb—body musculature, mc—cnidosac musculature, nu—nucleus, nc—NCs, ncp—NC cap, va—vacuoles, vc—vacuoles with chitinous spindles. Scale bars in µm.

## Data Availability

Unedited trees and morphological data for each species are provided as Supplementary material. Sets of unedited TEM, SEM, CLSM images for each studied species are available in Morphobank (http://morphobank.org/permalink/?P4334). Digital video of nudibranch feeding, and CLSM scanning stacks are available upon request from authors.
