# Supplementary material for: Nematocyst sequestration within the family Fionidae (Gastropoda: Nudibranchia) considering ecological properties and evolution
Source: Front Zool. 2022 Nov 16;19:29. doi: 10.1186/s12983-022-00474-9 (PMC9670572; doi:10.1186/s12983-022-00474-9)
Supplement: Supplementary file 1 — Additional file 1. Table S1. Number of specimens of each species used in this study. Abbreviations: spec = specimens. [file 12983_2022_474_MOESM1_ESM.docx]

Table S1. Number of specimens of each species used in this study. Abbreviations: spec = specimens.

| Species | Histological sections | TEM | CLSM | Radula morphology | Ecological observations |
| --- | --- | --- | --- | --- | --- |
| *Catriona columbiana* | 3 spec | 2 spec | 4 spec | 5 spec | underwater |
| *Cuthona nana* | 2 spec | 2 spec | 4 spec | 7 spec | lab |
| *Cuthonella osyoro* | 1 spec | - | 2 spec | 4 spec | underwater |
| *Cuthonella hiemalis* | 3 spec | 2 spec | 3 spec | 2 spec | lab |
| *Cuthonella marisalbi* | 1 spec | - | 4 spec | 4 spec | lab |
| *Diaphoreolis viridis* | 4 spec | 3 spec | 6 spec | 3 spec | lab |
| *Eubranchus odhneri* | 2 spec | 2 spec | 4 spec | 3 spec | - |
| *Eubranchus malakhovi* | 2 spec | 2 spec | 2 spec | 4 spec | - |
| *Eubranchus pallidus* | 1 spec | 1 spec | 3 spec | 1 spec | - |
| *Eubranchus rupium* | 6 spec | 4 spec | 7 spec | 4 spec | lab |
| *Tergipes tergipes* | 3 spec | 1 spec | 2 spec | 2 spec | lab |
| *Trinchesia ornata* | 1 spec | 1 spec | 3 spec | 1 spec | underwater |
| *Zelentia pustulata* | 4 spec | 2 spec | 7 spec | 5 spec | lab |
