# Supplementary figures and images for: Nematocyst sequestration within the family Fionidae (Gastropoda: Nudibranchia) considering ecological properties and evolution
Source: Front Zool. 2022 Nov 16;19:29. doi: 10.1186/s12983-022-00474-9 (PMC9670572; doi:10.1186/s12983-022-00474-9)

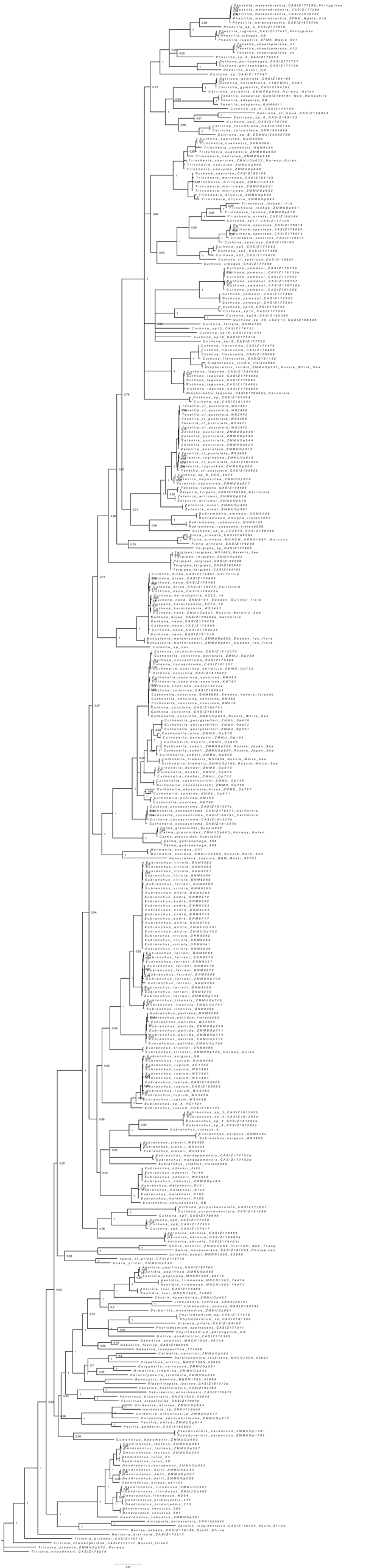

Supplement: Supplementary file 5 — Additional file 5. Data S1. Unedited maximum likelihood phylogenetic tree based on the concatenated dataset of three markers (COI+16S+H3) in NEWICK format. [file 12983_2022_474_MOESM5_ESM.pdf]

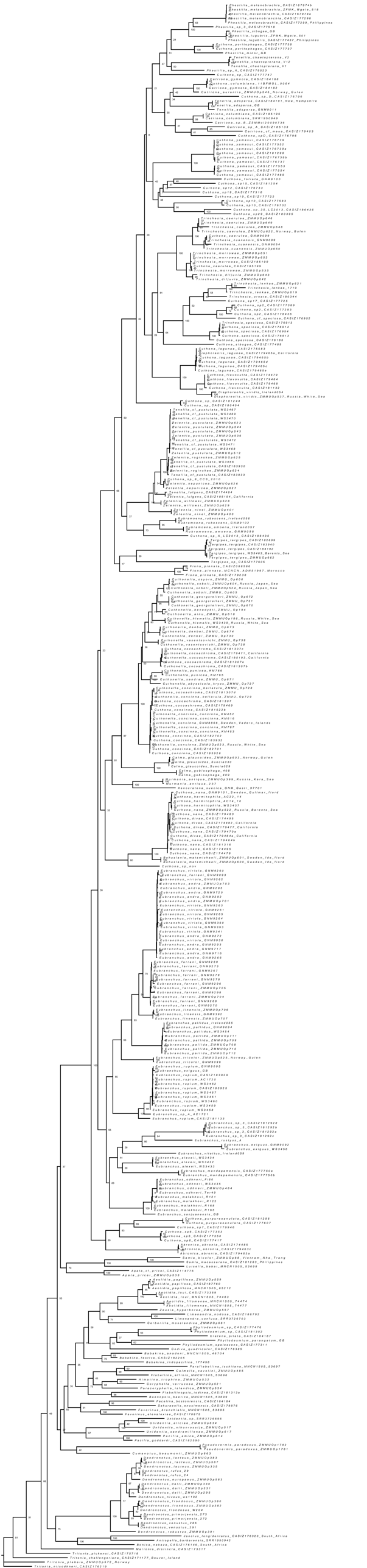

Supplement: Supplementary file 6 — Additional file 6. Data S2. Unedited Bayesian phylogenetic tree based on the concatenated dataset of three markers (COI+16S+H3) in NEWICK format. [file 12983_2022_474_MOESM6_ESM.pdf]

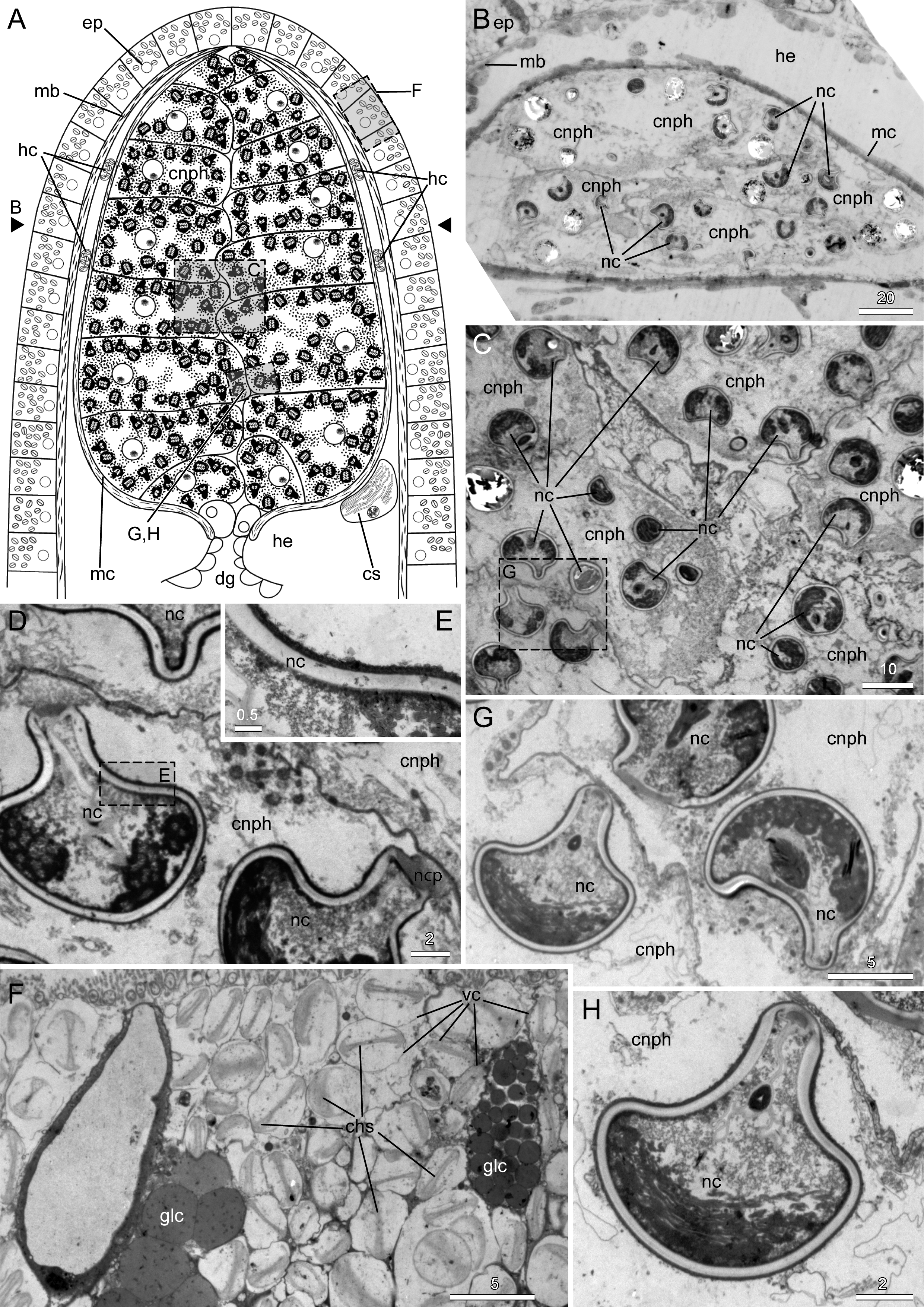

Supplement: Supplementary file 7 — Additional file 7. Figure S1. Catriona columbiana, cnidosac morphology. A—generalized scheme of cnidosac structure. B—cross-section through cnidophage zone. C—cnidophage zone. D—cnidophage cell membrane. E—NC wall. G—NCs in cnidophages. F—epidermis. H—NC within cnidophage. Abbreviations: cnph—cnidophage, chs—chitinous spindles, dg—digestive gland, ep—epithelium, glc—cells with granules, hc—cells with chitinous spindles, he—haemocoel, lu—lumen, mb—body musculature, mc—cnidosac musculature, nc—NCs, nu—nucleus, vc—vacuoles with chitinous spindles. Scale bars in µm. [file 12983_2022_474_MOESM7_ESM.tif]

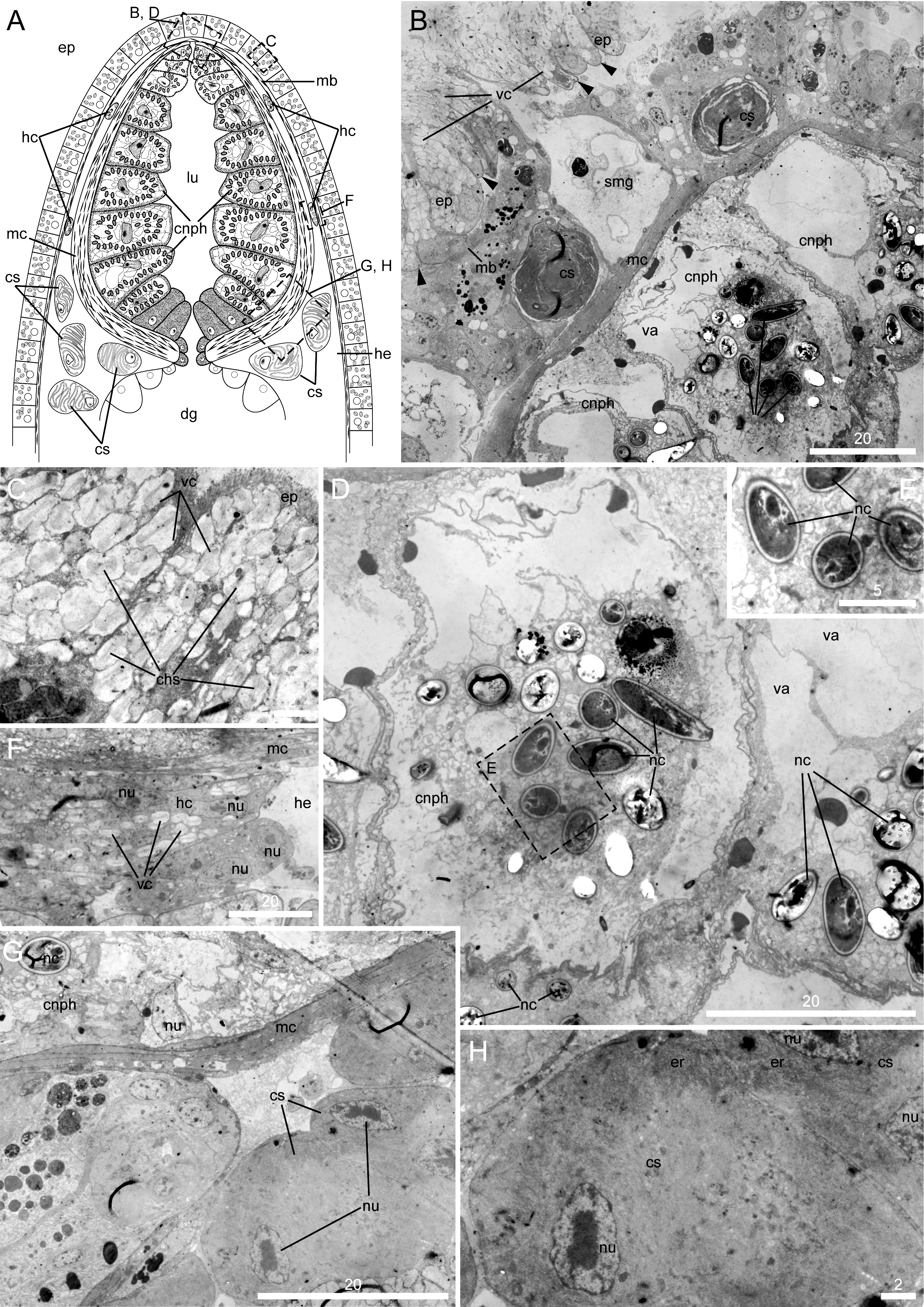

Supplement: Supplementary file 8 — Additional file 8. Figure S2. Cuthona nana, cnidosac morphology. A—generalized scheme of cnidosac structure. B—cnidopore zone, black arrowheads indicate basal laminae. C, F—epidermis. D—cnidophage. E—NCs within cnidophage. G—haemocoel with cellules speciale (cs). H—cellules speciale. Abbreviations: cnph—cnidophage, chs—chitinous spindles, cs—cellules speciale, dg—digestive gland, ep—epithelium, er—endoplasmic reticulum, hc—cells with chitinous spindles, he—haemocoel, lu—lumen, mb—body musculature, mc—cnidosac musculature, nu—nucleus, nc—NCs, smg—subepidemal mucus gland, va—vacuoles, vc—vacuoles with chitinous spindles. Scale bars in µm. [file 12983_2022_474_MOESM8_ESM.tif]

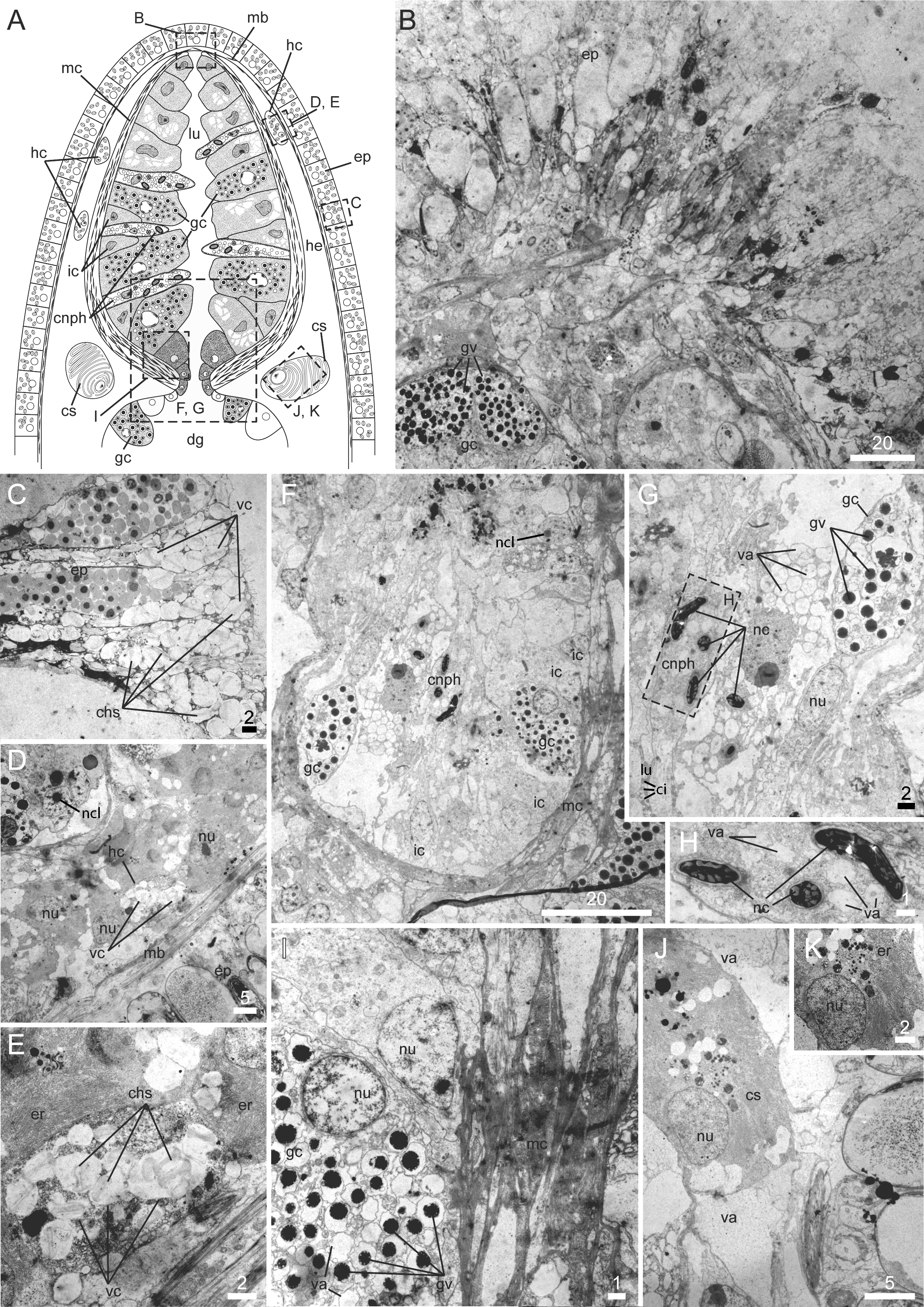

Supplement: Supplementary file 9 — Additional file 9. Figure S3. Cuthonella hiemalis, cnidosac morphology. A—generalized scheme of cnidosac structure. B, I—cnidopore zone. C—epidermis. D, E—haemocoel. F, G—cnidophage zone. H—nematocysts (nc) within cnidophages. J, K—cellules speciale. Abbreviations: cnph—cnidophage, ci—cilia, chs—chitinous spindles, cs—cellules speciale, dg—digestive gland, ep—epithelium, er—endoplasmic reticulum, gc—cells with granular compound, gv—vesicles with electron-dense granules, hc—cells with chitinous spindles, he—haemocoel, ic—interstitial cells, lu—lumen, mb—body musculature, mc—cnidosac musculature, nu—nucleus, ncl—nucleolus, nc—NCs, va—vacuoles, vc—vacuoles with chitinous spindles. Scale bars in µm. [file 12983_2022_474_MOESM9_ESM.tif]

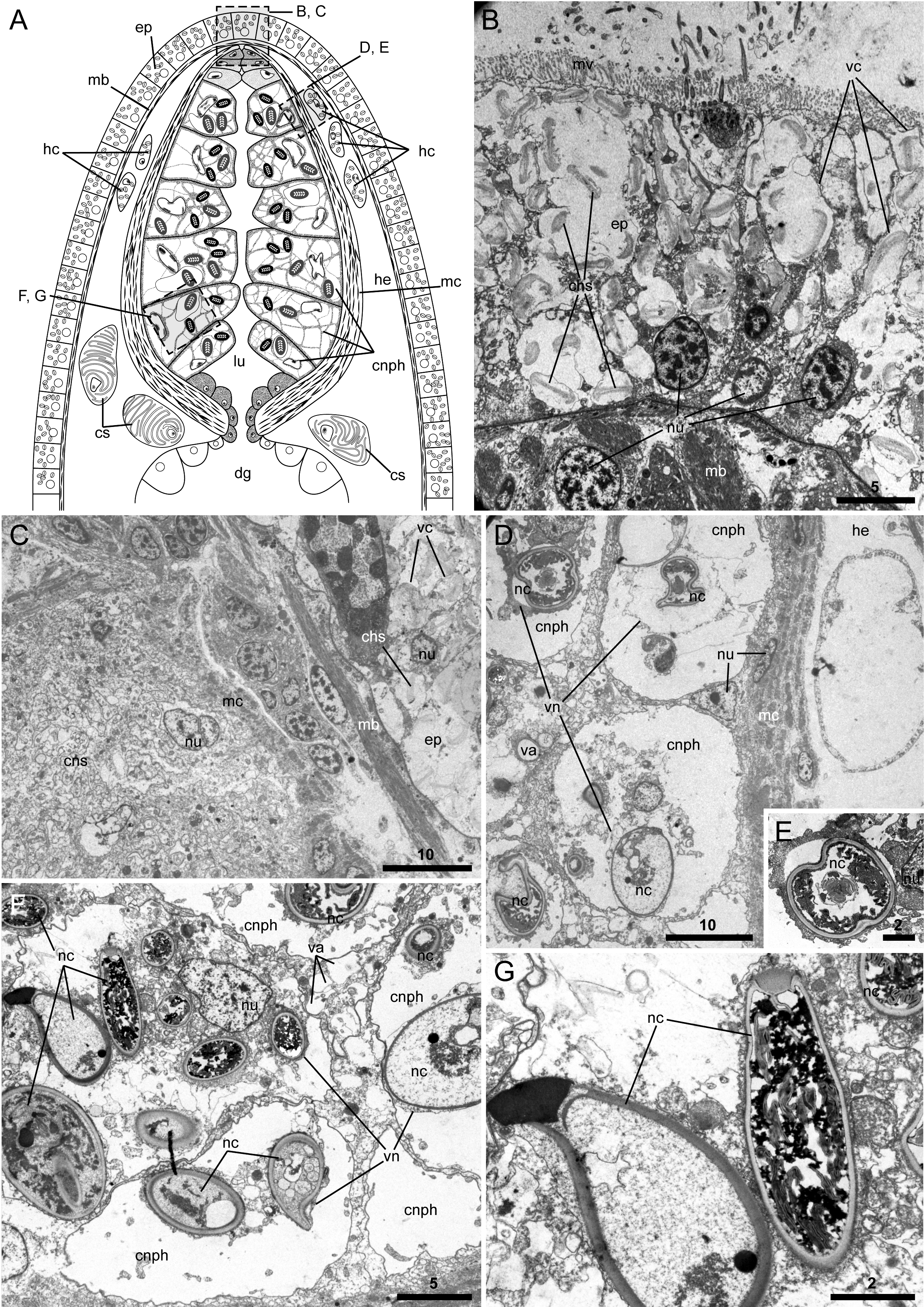

Supplement: Supplementary file 10 — Additional file 10. Figure S4. Diaphoreolis viridis, cnidosac morphology. A—generalized scheme of cnidosac structure. B, C—apical zone. D, E—cnidophage zone. F, G—NCs within cnidophages. Abbreviations: cnph—cnidophage, cns—cnidosac, chs—chitinous spindles, cs—cellules speciale, dg—digestive gland, ep—epithelium, hc—cells with chitinous spindles, he—haemocoel, lu—lumen, mb—body musculature, mc—cnidosac musculature, nu—nucleus, nc—NCs, va—vacuoles, vc—vacuoles with chitinous spindles. Scale bars in µm. [file 12983_2022_474_MOESM10_ESM.tif]

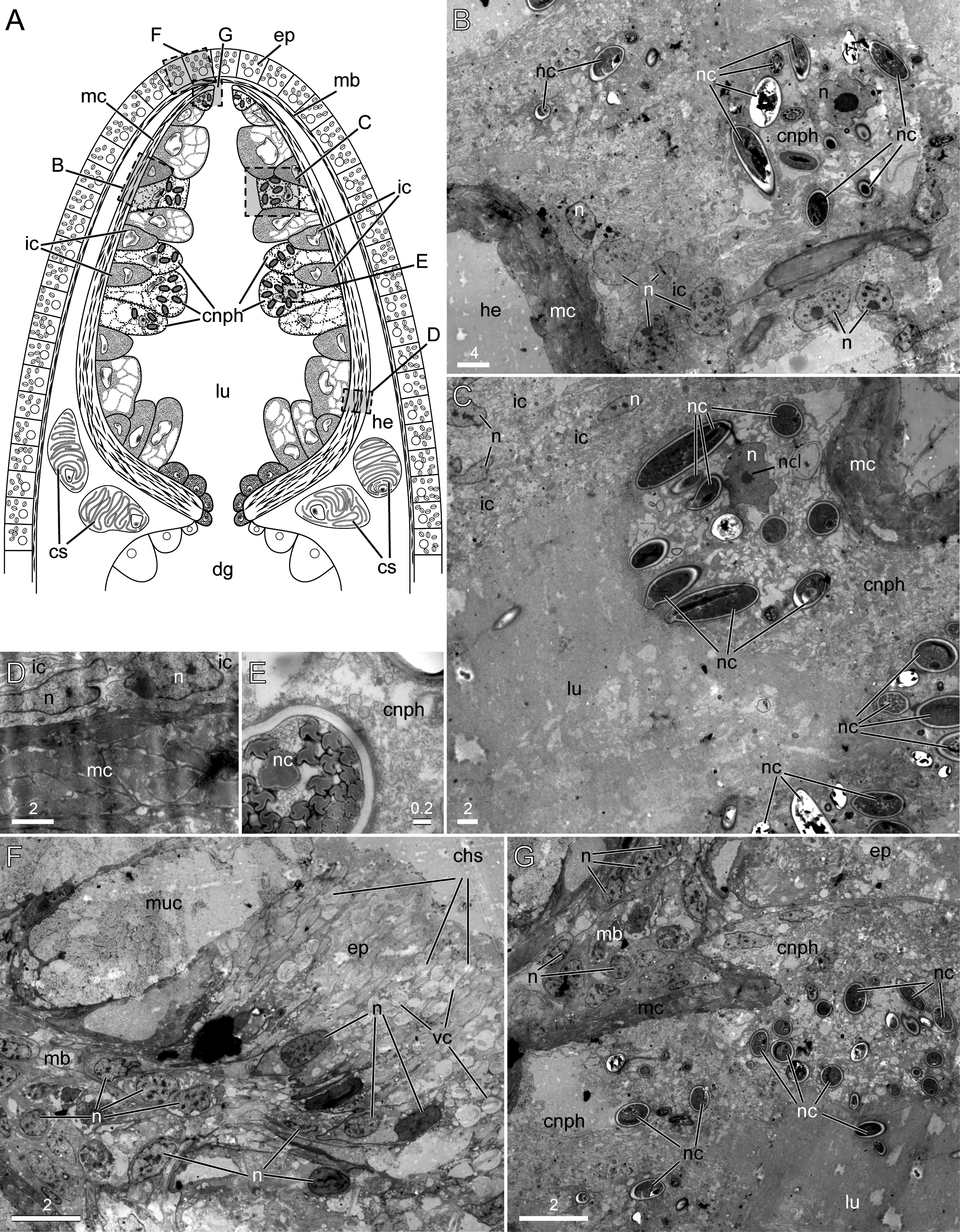

Supplement: Supplementary file 11 — Additional file 11. Figure S5. Eubranchus pallidus, cnidosac morphology. A—generalized scheme of cnidosac structure. B, C—cnidophage zone. D—cnidosac muscular wall. E—NCs within cnidophage. F—epidermis. G—cnidopore zone. Abbreviations: cnph—cnidophage, chs—chitinous spindles, cs—cellules speciale, dg—digestive gland, ep—epithelium, er—endoplasmic reticulum, ic—interstitial cells, he—haemocoel, lu—lumen, mb—body musculature, mc—cnidosac musculature, muc—mucous cell, n—nucleus, nc—NCs, ncl—nucleolus, vc—vacuoles with chitinous spindles. Scale bars in µm. [file 12983_2022_474_MOESM11_ESM.tif]

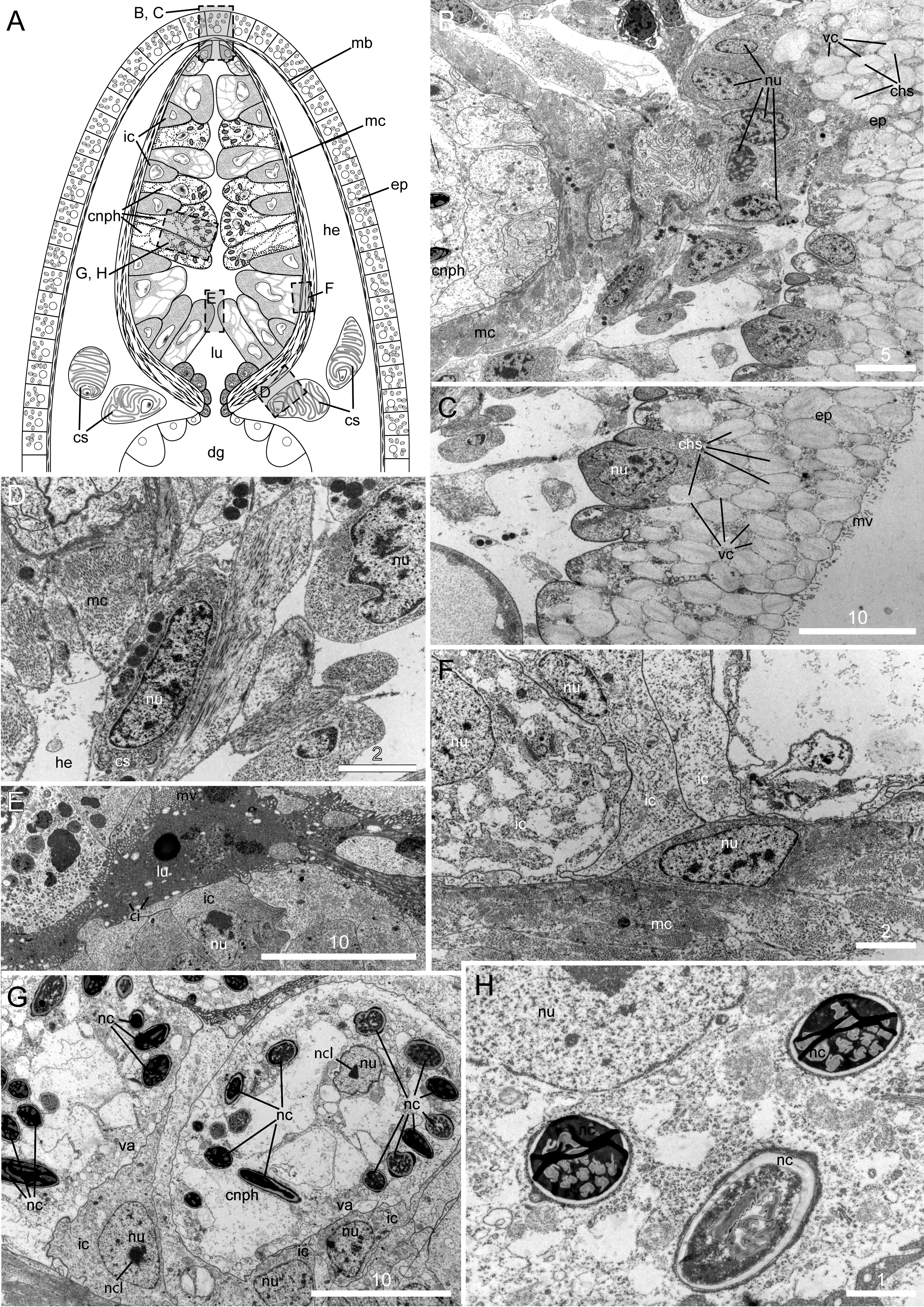

Supplement: Supplementary file 12 — Additional file 12. Figure S6. Eubranchus rupium, cnidosac morphology. A—generalized scheme of cnidosac structure. B—cnidopore zone. C—epidermis. D—haemocoel. E—cnidosac entrance. F, G—cnidophage zone. H—NCs within cnidophage. Abbreviations: ci—cilia, cnph—cnidophage, chs—chitinous spindles, cs—cellules speciale, dg—digestive gland, ep—epithelium, er—endoplasmic reticulum, ic—interstitial cells, he—haemocoel, lu—lumen, mb—body musculature, mc—cnidosac musculature, mv—microvilli, muc—mucous cell, nu—nucleus, nc—NCs, ncl—nucleolus, va—vacuoles, vc—vacuoles with chitinous spindles, vn—vacuoles with NCs. Scale bars in µm. [file 12983_2022_474_MOESM12_ESM.tif]

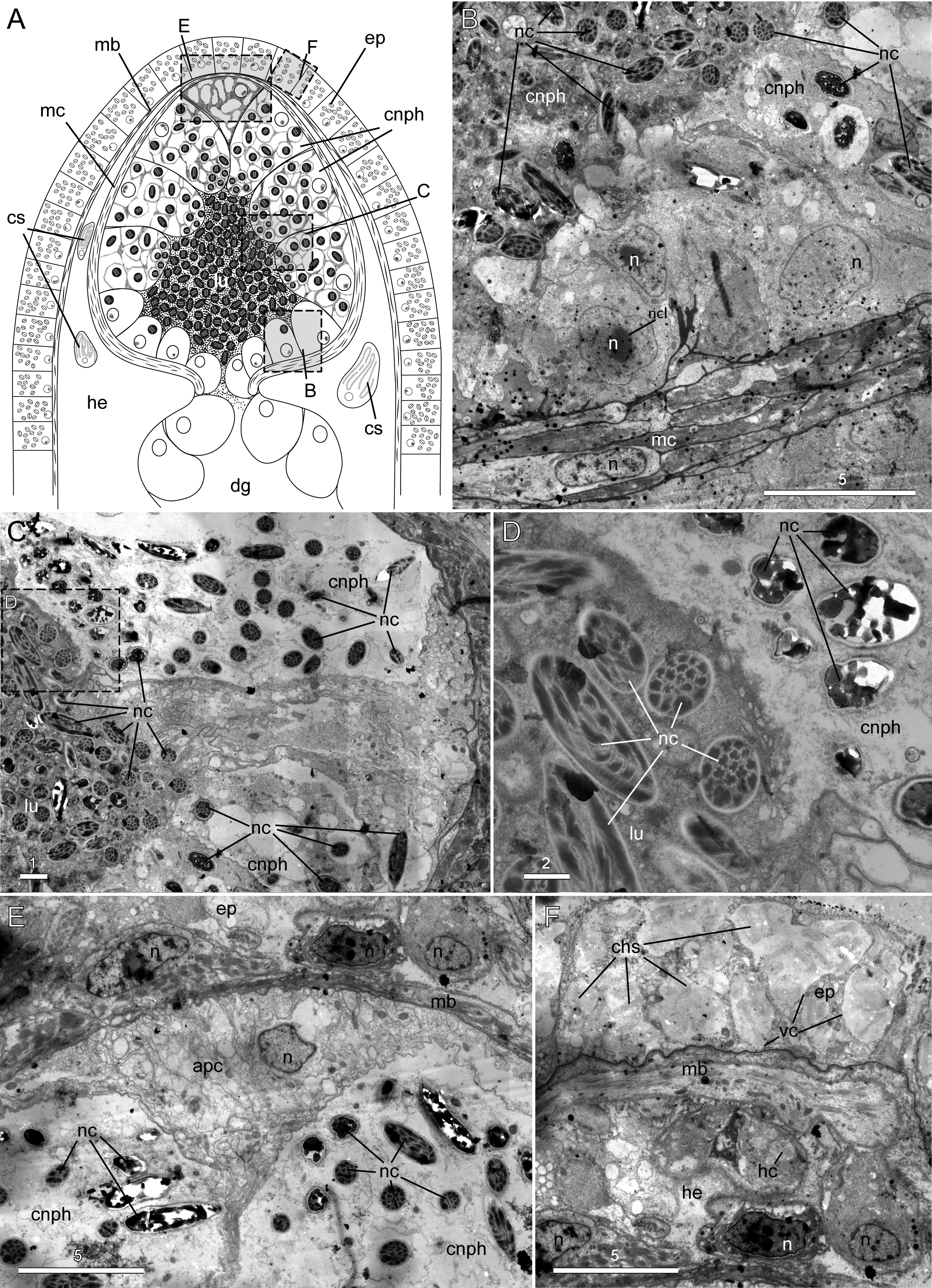

Supplement: Supplementary file 13 — Additional file 13. Figure S7. Tergipes tergipes, cnidosac morphology. A—generalized scheme of cnidosac structure. B—proliferation zone. C, D—cnidophage zone. E—cnidopore zone. F—epidermis. Abbreviations: apc—cell without NCs in cnidopore zone, cnph—cnidophage, cns—cnidosac, chs—chitinous spindles, cs—cellules speciale, dg—digestive gland, ep—epithelium, hc—cells with chitinous spindles, he—haemocoel, lu—lumen, mb—body musculature, mc—cnidosac musculature, n—nucleus, nc—NCs, ncl—nucleolus, va—vacuoles, vc—vacuoles with chitinous spindles. Scale bars in µm. [file 12983_2022_474_MOESM13_ESM.tif]

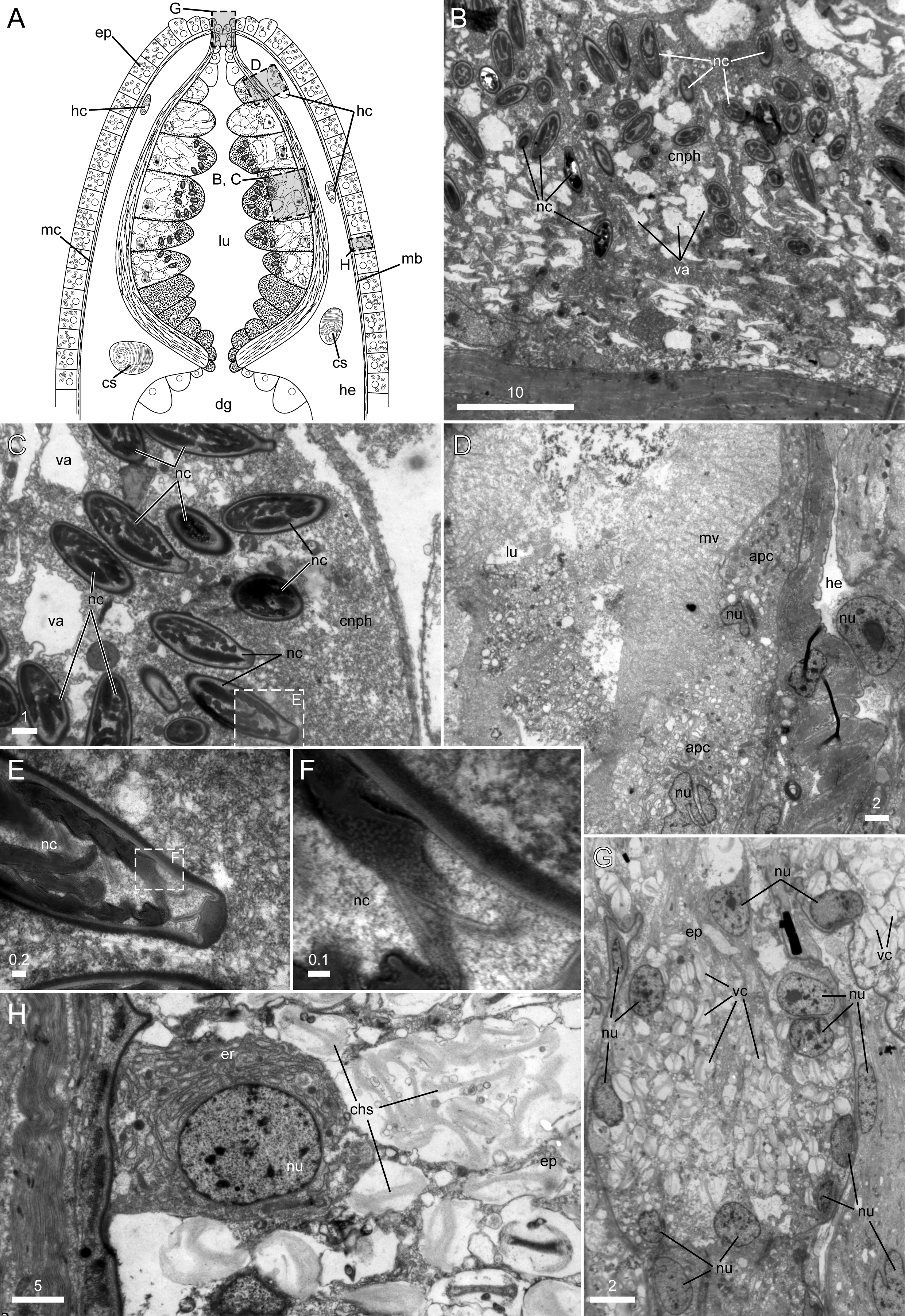

Supplement: Supplementary file 14 — Additional file 14. Figure S8. Trinchesia ornata, cnidosac morphology. A—generalized scheme of cnidosac structure. B, C—cnidophage zone. D—cnidopore zone. E, F—NCs within cnidophage. H—epidermis. G—epidermal invagination in cnidopore area. Abbreviations: apc—cells without NCs in cnidopore zone, cnph—cnidophage, cns—cnidosac, chs—chitinous spindles, cs—cellules speciale, dg—digestive gland, ep—epithelium, er—endoplasmic reticulum, hc—cells with chitinous spindles, he—haemocoel, lu—lumen, mb—body musculature, mc—cnidosac musculature, mv—microvilli, n—nucleus, nc—NCs, va—vacuoles, vc—vacuoles with chitinous spindles. Scale bars in µm. [file 12983_2022_474_MOESM14_ESM.tif]

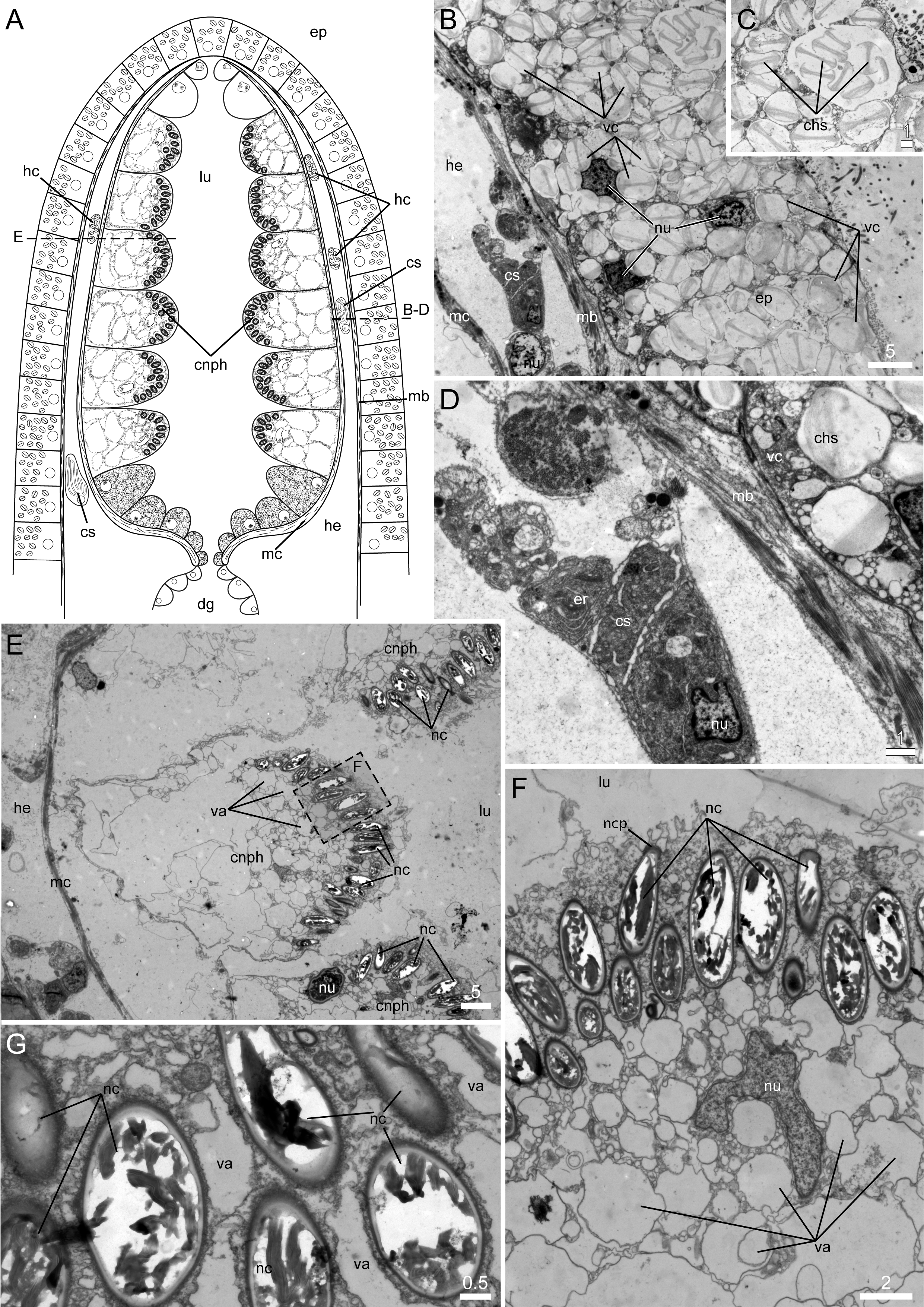

Supplement: Supplementary file 15 — Additional file 15. Figure S9. Zelentia pustulata, cnidosac morphology. A—generalized scheme of cnidosac structure. B, C—epidermis. D—haemocoel. E—cnidophage zone, cross-section. F—cnidophage. G—NCs within cnidophages. Abbreviations: cnph—cnidophage, chs—chitinous spindles, cs—cellules speciale, dg—digestive gland, ep—epithelium, er—endoplasmic reticulum, hc—cells with chitinous spindles, he—haemocoel, lu—lumen, mb—body musculature, mc—cnidosac musculature, nu—nucleus, nc—NCs, ncp—NC cap, va—vacuoles, vc—vacuoles with chitinous spindles. Scale bars in µm. [file 12983_2022_474_MOESM15_ESM.tif]
